# Comparative Evaluation of *Artemisia*-ZnO NPs and Chemically Synthesized ZnO NPs in Kefir: Effects on Quality and Probiotic Viability

**DOI:** 10.3390/foods15183271

**Published:** 2026-09-16

**Authors:** Noor Akhras, Hüseyin Bozkurt, Abuzer Çelekli

**Affiliations:** 1 Department of Biochemistry Science and Technology, Faculty of Arts and Science, Gaziantep University, Gaziantep 27310, Türkiye; 2 Department of Food Engineering, Faculty of Engineering, Gaziantep University, Gaziantep 27310, Türkiye; hbozkurt@gantep.edu.tr; 3 Department of Biology, Faculty of Art and Science, Gaziantep University, Gaziantep 27310, Türkiye; celekli.a@gmail.com

**Keywords:** *Artemisia*-ZnO NPs, kefir, probiotics, physicochemical properties, fermented dairy products, green synthesis

## Abstract

The growing interest in functional foods, such as kefir, has encouraged the investigation of green-synthesized nanoparticles as bioactive ingredients. This study investigated green-synthesized *Artemisia absinthium* L.-derived zinc oxide nanoparticles (*Artemisia*-ZnO NPs) incorporated into kefir at 0.25%, 0.50%, and 0.75% (*w*/*w*), with plain kefir and 0.25% chemically synthesized ZnO NPs as comparison formulations. Microbiological, physicochemical, and antioxidant properties were evaluated during 28 days of refrigerated storage at 4 °C. Structural characteristics of kefir samples were assessed using field emission scanning electron microscopy (FESEM), energy-dispersive X-ray spectroscopy (EDX), particle size distribution analysis, and Fourier transform infrared spectroscopy (FTIR). Sensory properties were evaluated at the end of storage using a nine-point hedonic scale. *Artemisia*-ZnO NPs significantly affected the microbiological, physicochemical, and sensory characteristics of kefir (*p* < 0.05). The 0.25% *Artemisia*-ZnO NP formulation exhibited higher DPPH radical-scavenging activity than plain kefir and 0.25% chemically synthesized ZnO NPs. This higher DPPH activity may reflect the combined effects of the *Artemisia*-ZnO NP system and the kefir matrix; however, the specific contribution of *Artemisia*-derived phytochemicals could not be established because their concentrations were not quantified in the final kefir, and differences in sample pH may also have influenced the DPPH response. Sensory evaluation showed that plain kefir received the highest scores, while higher *Artemisia*-ZnO NP concentrations reduced flavor and overall acceptability. Overall, the 0.25% *Artemisia*-ZnO NP formulation provided the most favorable balance of probiotic viability, physicochemical properties and sensory acceptability. However, further studies are required to evaluate zinc exposure, bioavailability, in vivo safety, and thujone content before its suitability as a functional ingredient can be established.

## 1. Introduction

The global demand for functional foods has increased significantly in recent years. At the same time, population growth and lifestyle changes have led to higher consumption of calorie-dense, nutrient-poor foods, contributing to major health problems such as obesity, cardiovascular diseases, diabetes, and neurodegenerative disorders [1]. As a result, consumers are increasingly seeking functional foods that provide not only basic nutrition but also additional health benefits. This growing interest has encouraged innovation in the food industry, particularly in the development of products that are low in calories, rich in vitamins and proteins, and free from synthetic additives [1,2].

Among functional foods, kefir has attracted considerable attention as a fermented dairy beverage with a unique nutritional composition and well-documented health-promoting properties [3]. Unlike conventional fermented milk products, kefir is produced by fermenting milk with kefir grains. These grains contain a complex microbial consortium of lactic acid bacteria, acetic acid bacteria, and yeasts responsible for the fermentation process. Their metabolic activity generates organic acids, peptides, vitamins, exopolysaccharides, and volatile compounds, which contribute to the characteristic flavor, texture, and functional properties of kefir [4,5].

Regular consumption of kefir has been associated with numerous health benefits, including modulation of the intestinal microbiota, enhancement of immune function, improvement of gastrointestinal health, antioxidant activity, potential antihypertensive, antimicrobial, anti-inflammatory, and cholesterol-lowering effects [4,5,6]. These biological activities are attributed to the metabolic activity of kefir microorganisms and the continuous production of bioactive compounds during fermentation, making kefir an attractive carrier for functional ingredients [3,4].

The physicochemical and sensory characteristics of kefir are closely related to the balance and activity of its microbial community [6]. Factors that influence microbial growth during fermentation and refrigerated storage can directly affect acidity, viscosity, texture, antioxidant capacity, flavor development, and overall consumer acceptance [6,7]. Consequently, earlier studies have focused on fortifying kefir with natural bioactive ingredients to enhance its nutritional and functional value while maintaining desirable quality attributes. These studies have demonstrated that the incorporation of functional ingredients, including encapsulated fruit extracts and plant-derived bioactive compounds, can enhance the antioxidant capacity, sensory properties, and overall functionality of kefir while maintaining acceptable physicochemical characteristics during storage [8,9]. Furthermore, maintaining a stable and metabolically active microbial community during refrigerated storage is important for preserving the functional quality and health-promoting properties of kefir, as storage-related microbial activity affects the composition and characteristics of the fermented product [10,11].

Parallel to these developments, nanotechnology has emerged as an important tool in food science [12]. Zinc oxide nanoparticles (ZnO NPs) have been widely studied in food systems due to their antimicrobial and functional properties [12,13]. In dairy products, several studies have reported their effects on microbial stability, texture, and shelf-life extension [14,15]. Green synthesis methods using plant extracts have gained attention as sustainable and eco-friendly alternatives to conventional approaches [16]. Plant extracts contain bioactive compounds such as flavonoids, phenolics, and terpenoids, which act as reducing and stabilizing agents during nanoparticle formation [16,17]. Compared with chemical synthesis, green synthesis reduces the use of toxic reagents and enhances nanoparticle stability through natural capping agents [18]. Our previous study investigated the green synthesis of ZnO NPs using *Artemisia absinthium* L. (*Artemisia*) extract and characterized their physicochemical properties and antimicrobial activity [19]. However, the potential application of these *Artemisia*-ZnO NPs in fermented dairy products has not been investigated. Although studies, particularly those conducted on yogurt, have reported that ZnO NPs can influence microbial viable counts, texture development, and shelf-life stability [14,15,20,21,22], information on green-synthesized ZnO NPs in kefir remains limited.

To the best of our knowledge, the application of *Artemisia absinthium* L.-derived ZnO NPs in kefir has not been previously reported. Their effects on probiotic viability and product quality during refrigerated storage also remain unexplored. This knowledge gap is relevant because the kefir matrix contains a complex microbial community and diverse fermentation-derived compounds that may influence nanoparticle behavior and, consequently, product quality during storage. Therefore, the present study evaluated the effects of *Artemisia*-ZnO NP incorporated into kefir at 0.25%, 0.50%, and 0.75% (*w*/*w*) on probiotic lactic acid bacterial viable counts, physicochemical properties and sensory characteristics. Plain kefir was used as the control, while kefir containing 0.25% (*w*/*w*) chemically synthesized ZnO NPs was included as a comparative formulation. In addition, antioxidant activity was evaluated at the 0.25% (*w*/*w*) concentration of *Artemisia*-ZnO NPs, against the plain kefir control and the 0.25% chemically synthesized ZnO NP formulation. A direct comparison between green-synthesized and chemically synthesized ZnO NPs was performed under identical experimental conditions to assess whether the synthesis approach was associated with differences in their effects within the kefir matrix. This comparison also allowed the effects of *Artemisia*-ZnO NPs to be assessed relative to ZnO NPs produced by conventional chemical synthesis while minimizing differences attributable to the food matrix and experimental conditions.

Furthermore, this study included structural characterization of kefir samples containing 0.25% green-synthesized *Artemisia*-ZnO NPs and 0.25% chemically synthesized ZnO NPs to examine their microstructural, elemental, particle size, and molecular characteristics. FESEM was employed to evaluate microstructural features and particle morphology, while EDX was used to determine elemental composition. Laser diffraction analysis was used to determine particle size distribution, and FTIR was conducted to assess changes in the molecular characteristics of the kefir samples following ZnO NP incorporation. Together with antioxidant activity measurements, these analyses provide complementary information on the structural and functional characteristics of nanoparticle-enriched kefir and help clarify the effects associated with *Artemisia*-ZnO NP incorporation.

## 2. Materials and Methods

### 2.1. Materials and Reagents

The aerial parts of *Artemisia absinthium* L. used for the green synthesis of ZnO NPs were collected from the Gaziantep region, Türkiye, and taxonomically identified by the Department of Biology and Food Engineering, Gaziantep University. Zinc nitrate hexahydrate [Zn(NO_3_)_2_·6H_2_O], sodium hydroxide (NaOH), ethanol (99.9%), and methanol (99.9%) were purchased from Merck (Darmstadt, Germany).

Raw cow’s milk was used for kefir production. Commercial single-use dehydrated kefir starter cultures (Sevdanem, Isparta, Türkiye) were used as the fermentation inoculum. MRS agar, M17 agar, peptone water, DPPH (1,1-diphenyl-2-picrylhydrazyl), phenolphthalein, potassium oxalate, formaldehyde solution (37%), and potassium bromide (KBr) were obtained from Sigma-Aldrich (Darmstadt, Germany). Petri dishes were supplied by ISOLAB (Wertheim, Germany). All chemicals and reagents used in this study were of analytical grade.

### 2.2. Methods

#### 2.2.1. Green and Chemical Synthesis of ZnO NPs

In our previous study, ZnO NPs were synthesized via a green co-precipitation method using *Artemisia* extract as a biogenic reducing and stabilizing agent. The resulting ZnO NPs were then characterized for their physicochemical and antimicrobial properties [19]. Briefly, *Artemisia*-ZnO NPs were obtained by dropwise addition of zinc nitrate hexahydrate solution into the plant extract under heating and alkaline conditions (NaOH, pH 8–12), followed by precipitation, washing, drying, and grinding to yield ZnO nanopowder [19]. For comparison, chemically synthesized ZnO NPs were prepared using the same co-precipitation procedure but without the addition of *Artemisia* extract, as described previously [19]. Both *Artemisia*-ZnO NPs and chemically synthesized ZnO NPs were subsequently incorporated into kefir under identical experimental conditions.

#### 2.2.2. Kefir Preparation and ZnO NP Incorporation

Kefir was prepared following the traditional method described in the literature [3,8]. Briefly, raw cow’s milk was pasteurized at 90 °C for 10 min to ensure microbiological safety and then cooled to 25 °C. A 5% (*w*/*w*) commercial freeze-dried kefir starter culture (SevDanem^®^, Danem Süt ve Süt Ürünleri Ltd. Şti., Isparta, Türkiye) was inoculated into the milk, which was subsequently fermented at 20–25 °C for 24 h. According to the manufacturer’s specifications, the starter culture contains a complex microbial consortium comprising lactic acid bacteria, bifidobacteria, acetic acid bacteria, and yeasts. Specific strain identifiers were not disclosed by the manufacturer. The dominant microbial groups include *Lactobacillus* spp. (20 × 10^9^ CFU/mL), *Lactococcus* spp. (20 × 10^9^ CFU/mL), *Lactobacillus acidophilus* (2 × 10^8^ CFU/mL), *Bifidobacterium animalis* subsp. *lactis* (2 × 10^7^ CFU/mL), and yeasts (2 × 10^4^ CFU/mL). The reported microbial composition includes *Lactobacillus kefiri*, *Lactobacillus kefiranofaciens* subsp. *kefiranofaciens*, *Lactobacillus kefiranofaciens* subsp. *kefirgranum*, *Lactobacillus parakefiri*, *Lactobacillus acidophilus*, *Lactobacillus casei*, *Lactobacillus reuteri*, *Lactobacillus bulgaricus*, *Lactobacillus helveticus*, *Lactobacillus fermentum*, *Leuconostoc mesenteroides*, *Lactococcus lactis*, *Streptococcus thermophilus*, *Bifidobacterium bifidum*, *Acetobacter pasteurianus*, *Kluyveromyces marxianus*, *Saccharomyces cerevisiae*, and *Kluyveromyces lactis*.

Following 24 h of fermentation, *Artemisia*-ZnO NPs were incorporated into kefir at concentrations of 0.25%, 0.50%, and 0.75% (*w*/*w*) and homogenized using a magnetic stirrer (MS300HS, MTops, Shanghai, China) for 40 min. Plain kefir, without NPs, was used as the control, while kefir supplemented with 0.25% (*w*/*w*) chemically synthesized ZnO NPs was included as a comparative formulation for determining the effects of *Artemisia*-ZnO NPs. All samples were stored at 4 °C for 28 days. During storage, probiotic bacterial counts, viscosity, color parameters, pH, titratable acidity, protein and total solids were evaluated on days 0, 1, 7, 14, 21, and 28.

#### 2.2.3. Characterization of ZnO-NPs Containing Kefir

The microstructural characteristics of kefir samples supplemented with 0.25% (*w*/*w*) *Artemisia*-ZnO NPs were examined at the beginning of the refrigerated storage period (Day 1) using a Zeiss SIGMA VP-500 field emission scanning electron microscope (Carl Zeiss AG, Berlin, Germany). Prior to analysis, kefir samples were freeze-dried and finely powdered. About 0.1 mg of each dried sample was mounted onto aluminum stubs using conductive carbon adhesive tape and sputter-coated with a thin layer of gold to improve surface conductivity. FESEM observations were performed to evaluate particle morphology, surface characteristics, and ZnO-NP aggregates within the kefir matrix.

Elemental composition analysis was conducted using an Oxford Instruments EDX detector (Oxford Instruments plc, Oxford, UK) attached to the FESEM instrument. The analysis was performed on the same 0.25% (*w*/*w*) *Artemisia*-ZnO NP-supplemented kefir formulation at the beginning of the refrigerated storage period (Day 1). The spectra were collected from different regions of the sample surface to determine the elemental constituents and assess the presence of ZnO NPs incorporated into the kefir matrix. The detected elements were expressed as relative weight percentages (WT%).

The particle size distributions of kefir samples supplemented with 0.25% (*w*/*w*) *Artemisia*-ZnO NPs and 0.25% (*w*/*w*) chemically synthesized ZnO NPs were determined at the beginning of the refrigerated storage period (Day 1) using a laser diffraction particle size analyzer (HORIBA LA-950, Horiba Ltd., Kyoto, Japan). Prior to measurement, the samples were diluted with distilled water and gently homogenized to ensure uniform dispersion and an appropriate optical concentration for analysis. Consequently, the reported particle size distributions represent the dispersed kefir suspensions under the measurement conditions rather than the undiluted kefir matrix. Measurements were performed at room temperature using the wet dispersion mode, in which the sample particles were suspended and analyzed in a liquid medium. The instrument was operated using an area-based particle size distribution, as specified by the instrument software program (HORIBA LA-950 for Windows, Version 5.2, HORIBA Ltd., Kyoto, Japan) (Distribution Base: Area). The particle size distribution was expressed as the differential area (%) distribution, while the cumulative distribution was reported as UnderSize (%). The characteristic particle diameters D10, D50 (median diameter), and D90 were obtained from the cumulative area-based distribution and represent the particle diameters below which 10%, 50%, and 90% of the cumulative particle-size distribution are found, respectively.

FTIR analysis was performed on kefir samples supplemented with 0.25% (*w*/*w*) *Artemisia*-ZnO NPs and 0.25% (*w*/*w*) chemically synthesized ZnO NPs at the beginning of the refrigerated storage period (Day 1) using a PerkinElmer Spectrum 100 spectrometer (PerkinElmer Inc., Berlin, Germany). Freeze-dried kefir samples were mixed with KBr in a 1:100 (*w*/*w*) sample-to-KBr ratio and analyzed using an FTIR spectrometer (PerkinElmer Inc., Berlin, Germany) over the spectral range of 4000–400 cm^−1^, with spectra recorded at a resolution of 4 cm^−1^ using 32 scans per sample. The obtained spectra were evaluated to identify characteristic functional groups of kefir constituents and ZnO-related vibrations and to assess potential changes following ZnO NP incorporation.

#### 2.2.4. Antioxidant Activity

Antioxidant activity was determined using the DPPH radical-scavenging assay with minor modifications [7,23]. Briefly, 2 g of kefir sample was mixed with 20 mL of methanol/water (70:30, *v*/*v*) and stirred at 20 ± 1 °C for 4 h in the dark. The mixture was then centrifuged at 3000 rpm for 12 min at 4 °C, and the supernatant was filtered through Whatman No. 2 filter paper. The obtained extract was used for antioxidant analysis. Subsequently, 0.25 mL of the extract was mixed with 0.18 mL of DPPH solution (10^−3^ M in methanol), and methanol was added to obtain a final volume of 3 mL. The reaction mixture was incubated in the dark at room temperature for 30 min. The absorbance was measured at 517 nm using a UV-1800 spectrophotometer (Shimadzu, Tokyo, Japan). The DPPH radical-scavenging activity was calculated using the following equation (Equation (1)):

(1) DPPH radical scavenging activity (%)=[1−(A sample/A control)]×100

where *A control* is the absorbance of the DPPH solution without sample and *A sample* is the absorbance in the presence of the kefir samples.

#### 2.2.5. Microbiological Analysis

In this study, the microbiological analysis was focused on lactic acid bacteria as the main functional probiotic indicators of kefir, in line with commonly used protocols in fermented dairy and nanotechnology-based food studies [14,15,20,21,22]. All samples were diluted tenfold using 0.1% (*w*/*v*) peptone water; an appropriate inoculation method was applied, and then incubated under conditions suitable for the target bacteria. Subsequently, bacterial colonies were counted. The bacterial counts of all kefir samples were determined after fermentation on days 0, 1, 7, 14, 21 and 28, with results being expressed as a logarithmic colony-forming unit (log CFU/mL).

**Figure 1:**
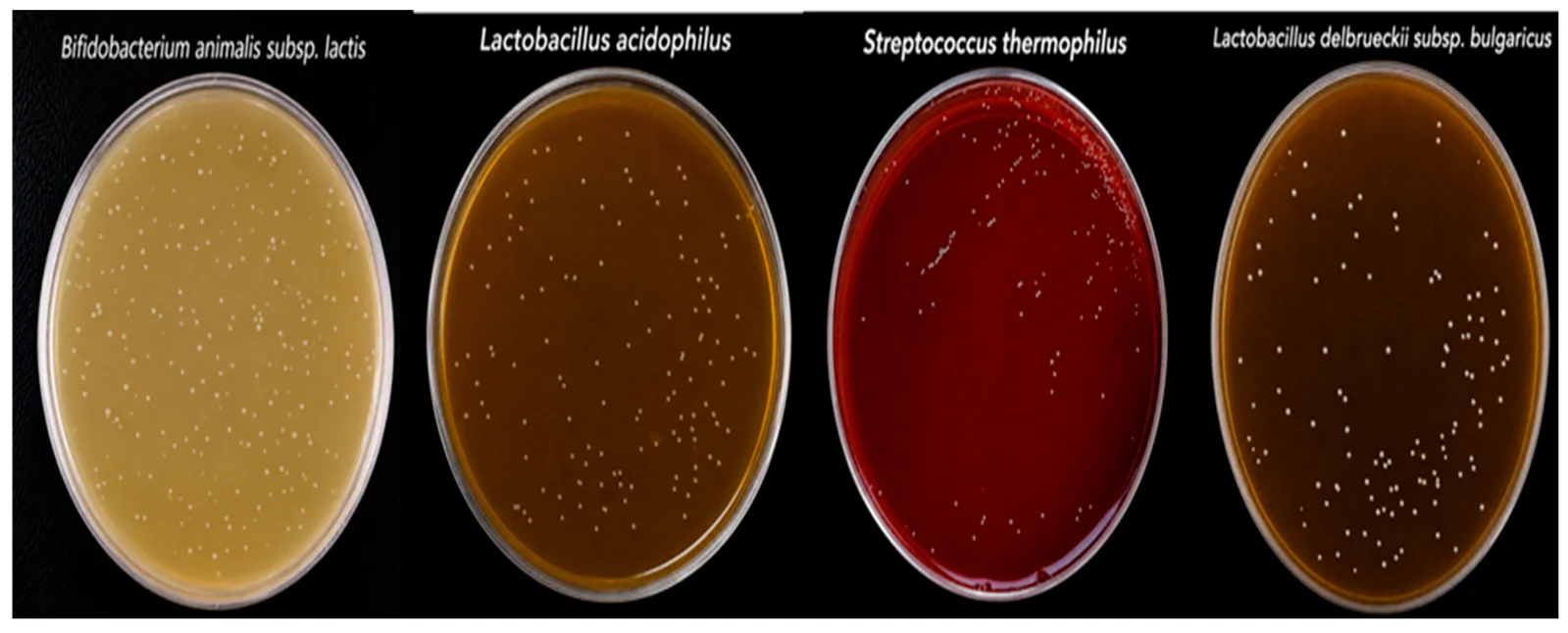
Representative colony morphology of the bacterial species used for the microbiological evaluation of kefir samples.

Bacterial growth was assessed following a previously described method [24]. *Lactobacillus acidophilus* was cultured on MRS agar with 1% maltose and incubated at 37 °C for 3 days. *Streptococcus thermophilus* was studied using the pour plate technique on M17 agar supplemented with 1% lactose and incubated at 45 °C for 24 h. Similarly, *Lactobacillus delbrueckii* subsp. *bulgaricus* was grown on MRS agar with 1% fructose and incubated at 45 °C for 3 days. Likewise, the viable counts of *Bifidobacterium animalis* subsp. *lactis* were evaluated on MRS agar and incubated at 45 °C for 72 h under anaerobic conditions using an anaerobic jar. These media are differential rather than absolutely selective. Presumptive enumeration of each bacterial species was achieved by combining carbohydrate-supplemented media under specific incubation conditions, including temperature, incubation time, and atmospheric conditions, following a previously described method [24]. Representative colony morphologies of the four bacterial species are shown in Figure 1.

#### 2.2.6. Physicochemical Analyses of Samples

The pH values were measured using a pH meter (WTW inoLab^®^ pH 7310, Xylem Analytics Germany Sales GmbH & Co. KG, WTW, Weilheim, Germany) on days 0, 1, 7, 14, 21 and 28 of storage at a temperature of 25 °C. Titratable acidity was determined by adding 3 drops of phenolphthalein to kefir samples and titrating with 0.1 N NaOH. Titratable acidity was expressed as % lactic acid and calculated using the following (Equation (2)) [25]:

(2) % lactic acid=v∗(0.009)∗100/m

where *v* is the volume of 0.1 N NaOH used for titration (mL), 0.009 is the lactic acid equivalent factor (g lactic acid per mL of 0.1 N NaOH), and *m* is the mass of the kefir sample (g).

The color parameters (L*, a*, and b*) of kefir samples were measured using a Hunter Lab ColorFlex (A60-1010-615 Model colorimeter, Hunter Associates Laboratory, Inc., Reston, VA, USA), a widely used instrument for objective color evaluation in dairy products. This method is suitable for kefir because it provides rapid, accurate, and reproducible measurements of lightness and color changes during storage [26]. Measurements were taken on days 0, 1, 7, 14, 21 and 28 to monitor color stability over time.

Apparent viscosity measurements were performed using a Brookfield DV3T™ viscometer (Brookfield Engineering Laboratories, Inc., Middleborough, MA, USA) at 30 rpm for 25 s using 150 mL of sample. Brookfield-type viscometers are widely used in dairy science, as they provide reproducible measurements under controlled shear conditions, allowing for reliable comparison of fermented milk systems. This method is considered appropriate for evaluating the rheological behavior of kefir and monitoring structural changes during fermentation and storage [26].

Total solids content was determined according to the AOAC method [27]. Three grams of kefir was placed in a pre-weighed and pre-dried glass dish and dried in a hot air oven at 105 °C until a constant weight was reached. The samples were then cooled in a desiccator before the final weight was recorded. This method is widely accepted for dairy products and is particularly suitable for kefir, as it accurately removes moisture while retaining all solid constituents, including proteins, fats, and minerals, thereby providing a reliable measure of total solids in fermented matrices. The total solid content was determined by the equations (Equations (3) and (4))

(3) Total solid %=100−moisture %

(4) Moisture %=[(ws−(w2−w1))/ws]×100

where w1 is the weight of the empty dish, w2 is the weight of the dish after drying, and ws is the weight of the sample.

The protein content of the samples was determined according to the Turkish standard method TS 1330 (2006) [28]. Briefly, 10 mL of the sample was transferred into a flask, and 0.4 mL of saturated potassium oxalate and 0.5 mL of 0.5% phenolphthalein solution were added. The mixture was shaken thoroughly and allowed to stand for 2 min. It was then titrated with 0.1 M NaOH until a faint pink color appeared. Subsequently, 2 mL of 37% formaldehyde solution, previously neutralized using phenolphthalein and 0.1 M NaOH, was added to the mixture. The solution was again titrated with 0.1 M NaOH until the original pink endpoint was reached, and the volume of NaOH used in this second titration (a) was recorded.

In parallel, a blank sample consisting of 2 mL of formaldehyde and 10 mL of distilled water was titrated under the same conditions, and the volume of NaOH consumed (b) was recorded. Protein content was calculated using Equation (5):

(5) Total protein %=1.7×(a−b)

where *a* represents the volume of NaOH (in mL) used for kefir and *b* represents the volume of NaOH (in mL) used for the blank (water).

This method, based on formol titration, is particularly suitable for fermented dairy products such as kefir because it quantifies amino nitrogen derived not only from intact proteins but also from peptides and free amino acids formed during fermentation. The addition of potassium oxalate minimizes interference from calcium ions, while the initial neutralization step reduces the effect of organic acids, ensuring greater accuracy in complex matrices [26,27].

#### 2.2.7. Sensory Analysis

The study protocol was approved by the Research Ethics Board of Gaziantep University, Faculty of Science and Engineering (Protocol No. 025; Approval No. 648544), and written informed consent was obtained from all participants prior to sensory evaluation. Sensory evaluation was conducted at the end of the 28-day refrigerated storage period to assess the sensory attributes of the kefir samples. The analysis involved 18 assessors selected from among the staff and postgraduate students of the Department of Food Engineering, Gaziantep University. The evaluation was performed in a controlled sensory laboratory environment under standardized lighting and temperature conditions (22 ± 2 °C), in accordance with ISO 8589:2007 [29]. All samples were evaluated under blind conditions, coded with random two-digit numbers, and presented in a randomized order to minimize bias. Each panelist received a 100 mL portion of each kefir sample served in a neutral white plastic cup. Panelists evaluated appearance, color, odor, flavor, and overall acceptability using a 9-point hedonic scale [30], where 1 = dislike extremely and 9 = like extremely. Between evaluations, panelists cleansed their palates with water and plain crackers to avoid residual flavor effects. The collected sensory data were statistically analyzed to determine differences in sensory acceptability among the kefir formulations.

#### 2.2.8. Statistical Analysis

Statistical analyses were performed to evaluate the effects of (0.25%, 0.50%, and 0.75%) *Artemisia*-ZnO NPs, 0.25% chemically synthesized ZnO NPs, and refrigerated storage on kefir samples. Data were analyzed using one-way ANOVA with SPSS version 19.0 (SPSS Inc., Chicago, IL, USA). Day 0 represented the baseline measurement obtained after completion of the fermentation period (24 h) and before ZnO NP incorporation; therefore, the identical Day 0 values shown for the treatment groups represent a common pre-treatment baseline. Treatment effects were evaluated based on measurements obtained after NP incorporation. A single production batch was divided into the respective treatment groups and followed throughout the 28 days of refrigerated storage. Measurements at each sampling time were performed in triplicate, representing analytical replicates rather than independent production replicates. Differences among treatments were evaluated at each post-treatment sampling day, while changes during refrigerated storage were assessed within each treatment. Because the same production batch was evaluated across storage times, observations were not statistically independent; a repeated-measures or mixed-effects model was not applied, which represents a limitation of the analysis. Duncan’s multiple range test was used for post hoc comparisons at *p* < 0.05. Results are expressed as means ± standard deviation (SD).

## 3. Results and Discussion

### 3.1. Characterization of ZnO-NP-Containing Kefir

**Figure 2:**
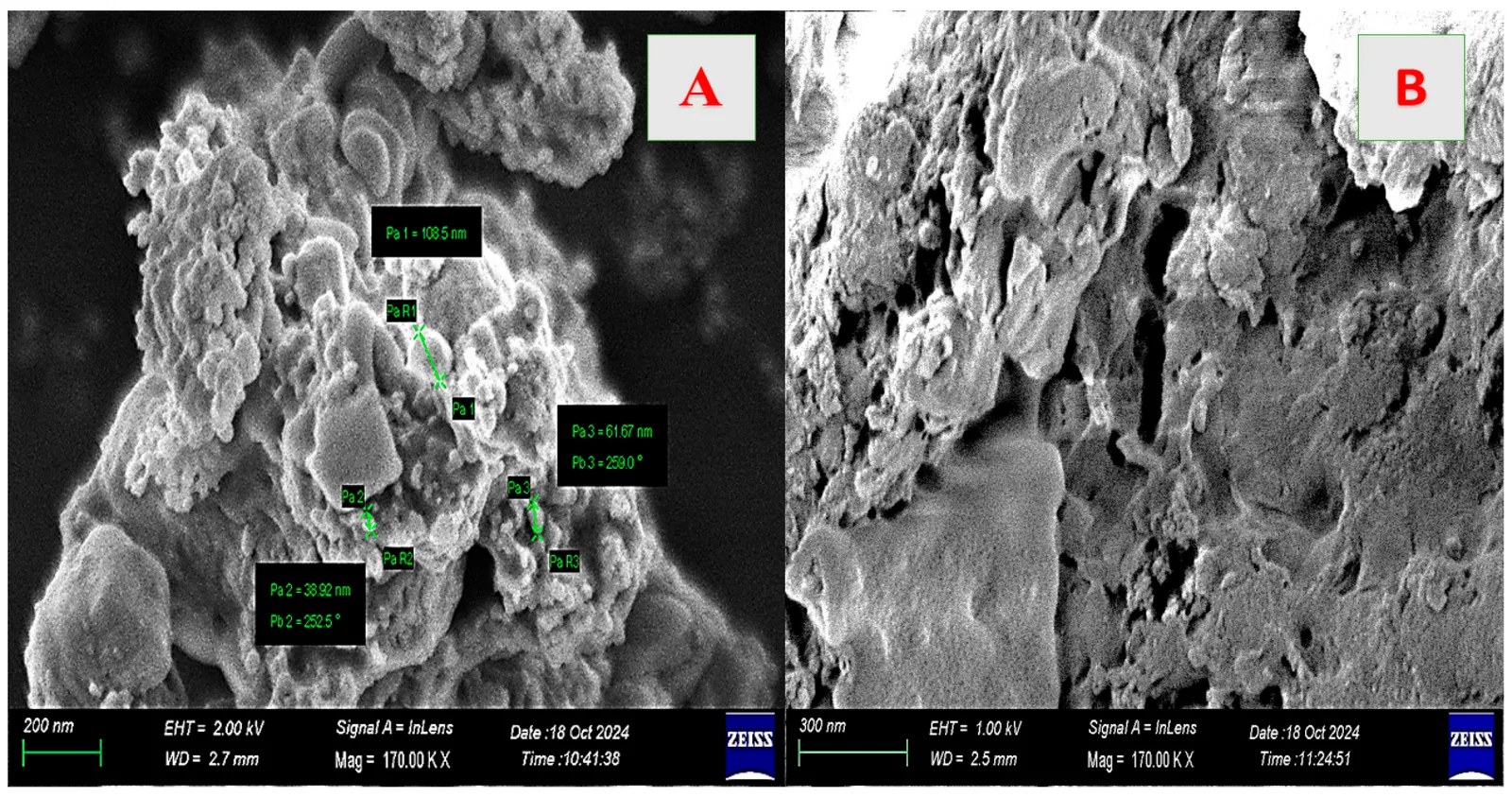
FESEM micrographs of kefir samples containing 0.25% (*w*/*w*) *Artemisia*-ZnO NPs (**A**) and 0.25% (*w*/*w*) chemically synthesized ZnO NPs (**B**) at the beginning of the refrigerated storage period (Day 1), showing aggregate-like structures within the fermented matrix.

The FESEM micrographs of kefir supplemented with 0.25% *Artemisia*-ZnO NPs and 0.25% chemically synthesized ZnO NPs showed noticeable aggregate-like structures within the ZnO-containing fermented matrix (Figure 2). These structures appeared irregular and densely packed, with heterogeneous sizes, forming submicron-scale clusters rather than a uniformly distributed morphology. Such aggregation-like features may be associated with physicochemical interactions between ZnO NPs and kefir constituents, particularly milk proteins and polysaccharides, which can influence particle dispersion and promote cluster formation within the fermented network. Similar interactions between ZnO NPs and milk protein matrices have been reported previously [31], while the dense protein–polysaccharide network of kefir may further contribute to particle entrapment and aggregation. Kefir containing 0.25% chemically synthesized ZnO NPs also showed similar aggregate-like structures within the fermented matrix, suggesting that ZnO-containing formulations may undergo comparable interactions with kefir constituents. Overall, the FESEM observations indicated the presence of aggregate-like structures within the ZnO-containing kefir matrices, but their specific composition cannot be definitively assigned to ZnO based on FESEM images alone.

**Figure 3:**
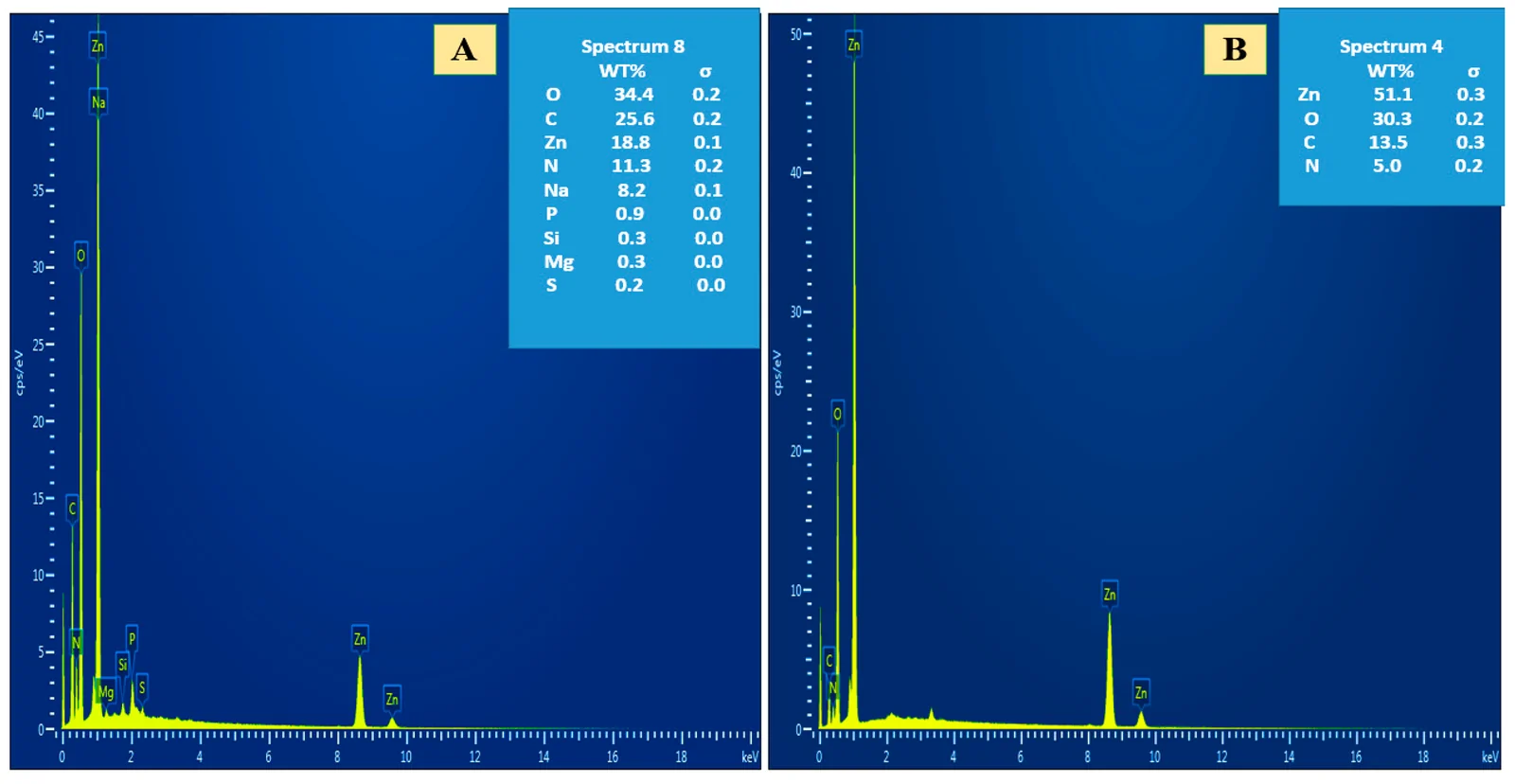
EDX spectra of kefir samples containing 0.25% (*w*/*w*) *Artemisia*-ZnO NPs (**A**) and 0.25% (*w*/*w*) chemically synthesized ZnO NPs (**B**), at the beginning of the refrigerated storage period (Day 1), showing Zn and O signals together with elements detected from the kefir matrix.

EDX analysis of kefir samples containing 0.25% (*w*/*w*) *Artemisia*-ZnO NPs and 0.25% (*w*/*w*) chemically synthesized ZnO NPs revealed Zn and O among the major detected elements, together with C, N, and other minor elements (Figure 3). The detected Zn and O signals may be associated with the added ZnO formulations, although their specific origin cannot be definitively established by EDX alone. The relatively high carbon and oxygen contents in both samples may be attributed to the organic constituents of the kefir matrix, including proteins, lipids, carbohydrates, and exopolysaccharides. The presence of Zn in both 0.25% ZnO NP-containing formulations is consistent with the elemental composition expected following ZnO NP incorporation but does not by itself confirm that the detected Zn originated exclusively from the added nanoparticles. The two samples showed broadly similar elemental profiles, suggesting comparable elemental characteristics under the analyzed conditions.

**Figure 4:**
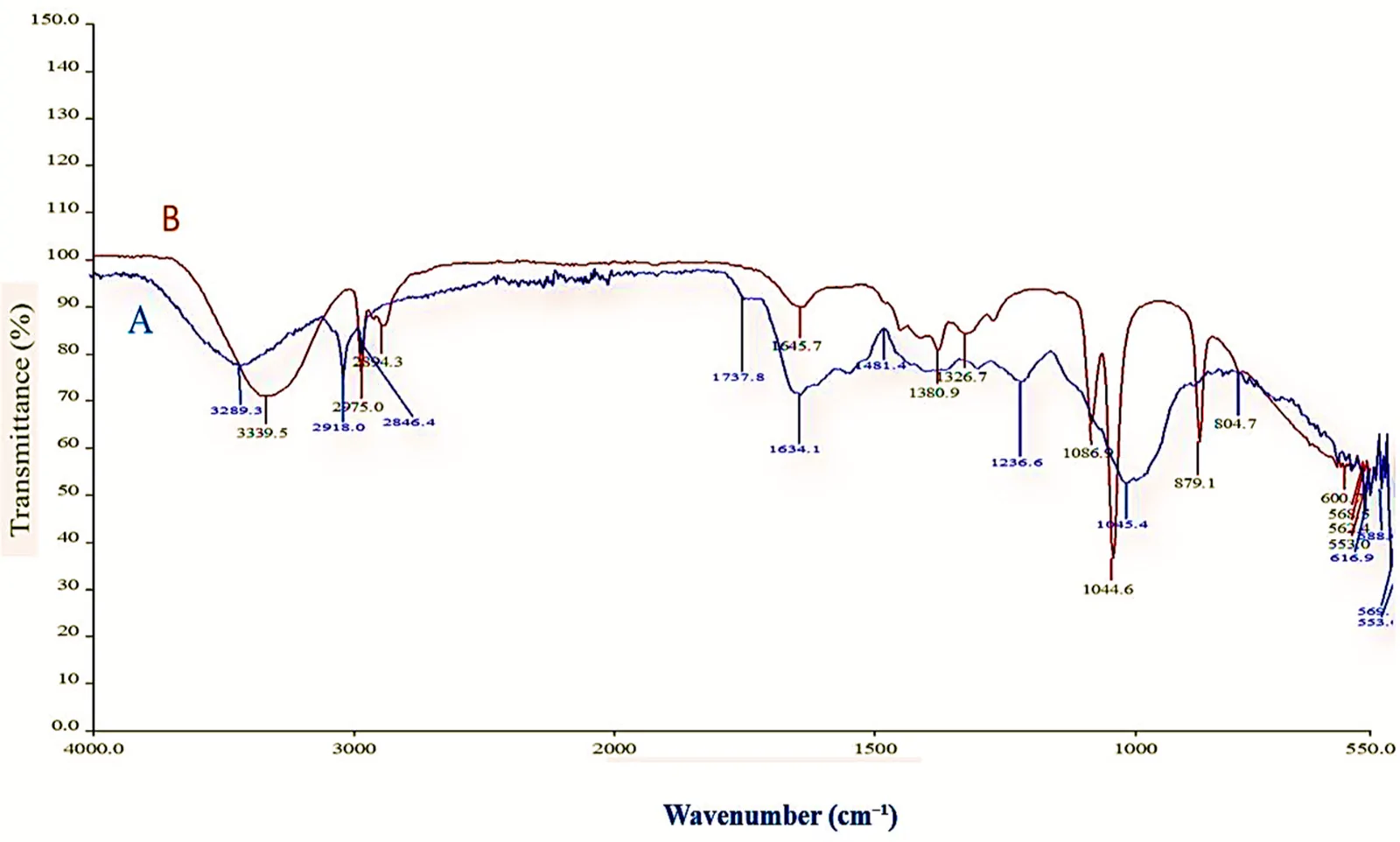
FTIR spectra of kefir containing 0.25% (*w*/*w*) *Artemisia*-ZnO NPs (A) and 0.25% (*w*/*w*) chemically synthesized ZnO NPs (B) at the beginning of the refrigerated storage period (Day 1).

FTIR analysis of kefir samples containing 0.25% *Artemisia*-ZnO NPs and 0.25% chemically synthesized ZnO NPs revealed the characteristic absorption bands of kefir constituents together with spectral features that may be associated with the ZnO-containing formulations (Figure 4). The broad absorption bands observed at approximately 3289 cm^−1^ (A) and 3339 cm^−1^ (B) were possibly associated with O–H and N–H stretching vibrations originating from water molecules, proteins, polysaccharides, and other hydrogen-bonded components present in kefir. The slightly lower wavenumber observed in the *Artemisia*-ZnO NP sample may reflect differences in the molecular environment within the O–H/N–H stretching region. Similar broad bands in the region of 3200–3400 cm^−1^ have been reported for fermented dairy products, with these bands being generally associated with protein and polysaccharide structures [15].

The absorption bands around 2918–2975 cm^−1^ may be attributed to C–H stretching vibrations of aliphatic groups present in kefir constituents. These bands were observed in both samples, indicating broadly similar spectral features in this region between the two kefir formulations. The bands observed at 1634 cm^−1^ (A) and 1646 cm^−1^ (B) are characteristic of amide I vibrations arising mainly from the C=O stretching of milk proteins. The slight shift between the two samples may reflect changes in the local molecular environment of these functional groups following ZnO NP incorporation. Similar shifts in amide bands have been previously reported following interactions between ZnO NPs and dairy proteins [31], which provides a possible context for the spectral differences observed here.

Additional bands detected at approximately 1381 and 1327 cm^−1^ in the chemically synthesized ZnO NP sample and at 1237 cm^−1^ in the *Artemisia*-ZnO NP sample may be attributed to C–N stretching and amide III vibrations associated with protein-containing constituents. The stronger intensity and broader distribution of these bands in the *Artemisia*-ZnO NP sample could reflect the presence of surface-associated compounds derived from *Artemisia*, which may contribute additional oxygen-containing functional groups to the nanoparticle surface.

The prominent absorption bands in the region of 1045–1087 cm^−1^ are mainly associated with C–O and C–O–C stretching vibrations of lactose, polysaccharides, and other kefir constituents. The differences observed in this region could reflect changes in the molecular environment of these components following ZnO NP incorporation, as reported previously for protein–nanoparticle interactions in dairy systems [31]. Such interactions are relevant as the dissolution behavior, matrix interactions, and fate of ZnO NPs as a food additive can influence their functionality and stability in complex food systems [32]. The low-frequency absorption bands below 700 cm^−1^ observed in both spectra may be associated with Zn–O-related vibrations. The *Artemisia*-ZnO NP sample exhibited more pronounced features in this region, reflecting differences in the matrix environment and the contribution of plant-derived surface compounds. Similar Zn–O absorption bands in the range of 450–650 cm^−1^ have been reported for both green-synthesized and chemically synthesized ZnO NPs in previous studies [33,34]. However, because the FTIR measurements were conducted on the complex kefir matrix, the resulting spectra represent the combined vibrational contributions of its various constituents. Consequently, the observed bands cannot be attributed specifically to ZnO and should not be considered definitive evidence of ZnO NP incorporation in the kefir matrix.

Overall, the FTIR findings were consistent with the FESEM and EDX observations, providing additional information on the ZnO-containing kefir formulations. The observed shifts in hydroxyl-, amide-, and carbohydrate-related bands were associated with changes in the spectral environment of proteins and polysaccharides within the fermented matrix. Notably, the *Artemisia*-ZnO NP sample exhibited more pronounced spectral modifications, which may reflect differences in the matrix environment and the surface characteristics of the phytochemical-coated nanoparticles. These observations are consistent with findings from earlier studies showing that phytochemicals associated with green-synthesized ZnO NPs can act as surface capping agents and contribute to nanoparticle stability and interactions with biopolymer-based food matrices [33,34].

**Figure 5:**
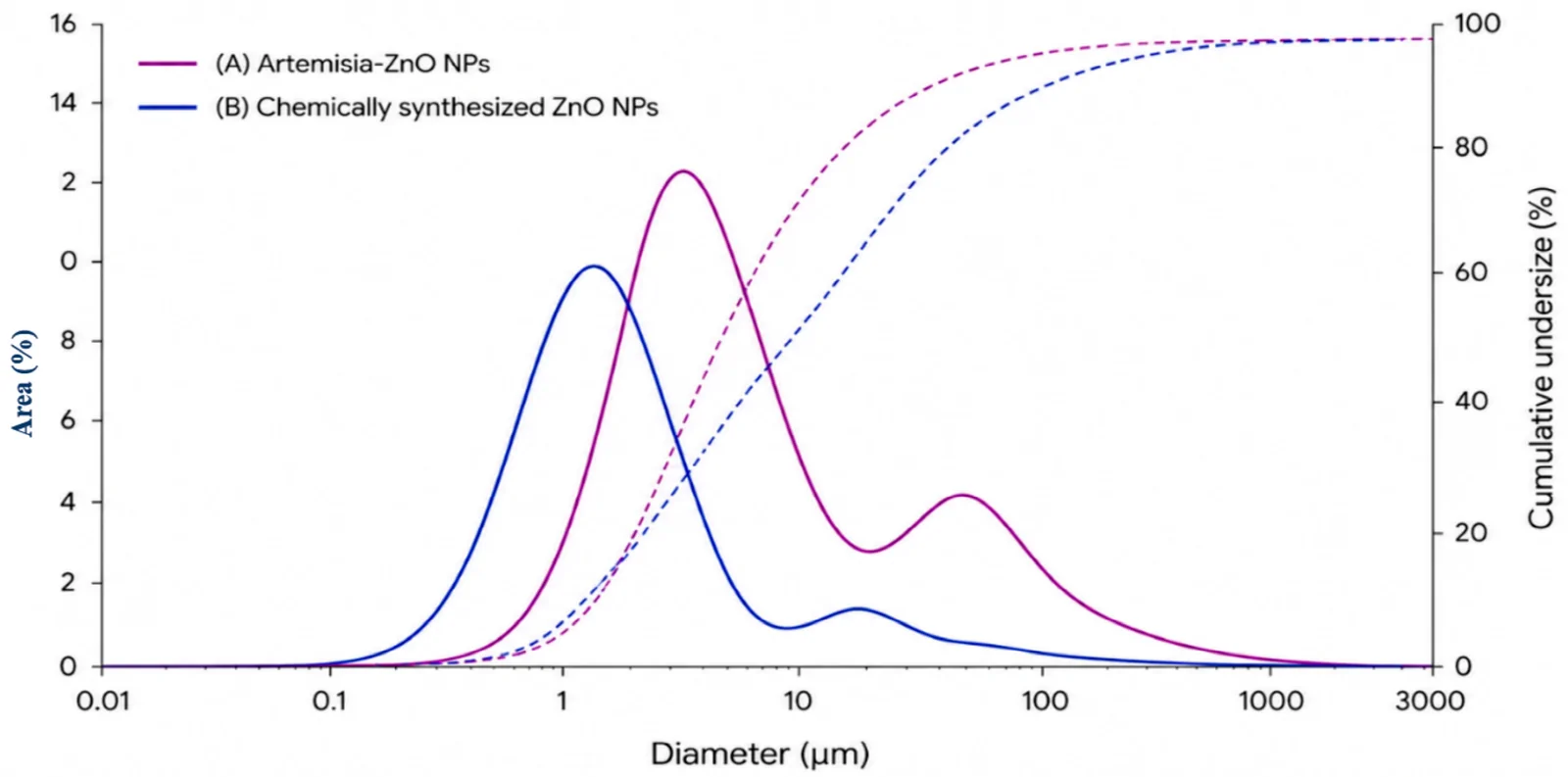
Particle size distribution and cumulative undersize curves of kefir samples containing (A) 0.25% *Artemisia*-ZnO NPs and (B) 0.25% chemically synthesized ZnO NPs at the beginning of the refrigerated storage period (Day 1). Solid lines represent area (%) on the left Y-axis and dashed lines represent cumulative undersize (%) on the right Y-axis.

The laser diffraction analysis demonstrated differences in the area-based particle size distributions of kefir supplemented with 0.25% *Artemisia*-ZnO NPs and 0.25% chemically synthesized ZnO NPs (Figure 5). The *Artemisia*-ZnO NP formulation exhibited a larger mean particle size (2.44 µm) and median diameter (1.16 µm) than the chemically synthesized ZnO NP formulation (1.75 and 0.67 µm, respectively), which may be consistent with the presence of larger particle assemblies within the kefir matrix. Similarly, the D90 value was slightly higher for the *Artemisia*-ZnO NP sample (4.99 µm) than for the chemically synthesized ZnO NP sample (4.71 µm), consistent with a modest difference in the upper particle size fraction. For D10, the chemically synthesized ZnO NP formulation exhibited a smaller D10 value (0.26 µm versus 0.38 µm), which may indicate a relatively greater proportion of finer particles (Supplementary Figures S1 and S2). These differences could be related to surface-associated compounds originating from the green synthesis process and their interactions with milk proteins and other colloidal components of kefir [33,34,35]. These interactions are consistent with differences in particle aggregation compared with chemically synthesized ZnO NPs. Furthermore, the larger particle sizes observed in the kefir samples likely reflect the formation or persistence of ZnO NP aggregates under the measurement conditions and their interactions with components of the food matrix, rather than the primary size of individual ZnO NPs.

### 3.2. Antioxidant Activity Results

The antioxidant activity of the kefir samples differed across the tested treatments and storage times (Table 1). Kefir containing 0.25% *Artemisia*-ZnO NPs exhibited the highest antioxidant activity, reaching 38.52 ± 0.16% on Day 1 and remaining comparatively high at 36.82 ± 0.37% on Day 28. Kefir containing 0.25% chemically synthesized ZnO NPs showed intermediate antioxidant activity, with a value of 27.20 ± 0.21% on Day 1, whereas plain kefir exhibited the lowest values during storage. DPPH radical-scavenging activity gradually decreased during refrigerated storage.

At each storage interval, the 0.25% *Artemisia*-ZnO NP group exhibited significantly higher antioxidant activity than the 0.25% chemically synthesized ZnO NP and control groups (*p* < 0.05). The higher antioxidant activity observed in the *Artemisia*-ZnO NP treatment may reflect differences in the overall formulation, including compounds associated with the *Artemisia*-derived nanoparticle system. Previous characterization of *Artemisia*-ZnO NPs demonstrated the presence of phenolic and flavonoid compounds in the nanoparticle system [19]. However, phytochemical content was not quantified in the kefir samples; therefore, the specific contribution of these compounds to the measured DPPH activity cannot be established.

Additionally, it should be noted that the DPPH assay may be influenced by sample pH. Differences in acidity among the kefir formulations could therefore partially affect the measured radical-scavenging activity. Previous studies have reported that DPPH responses can vary according to the pH conditions of the reaction system [36]. Therefore, some of the observed differences among treatments may reflect variations in sample acidity in addition to differences in antioxidant constituents. Nevertheless, the consistently higher antioxidant activity observed in the 0.25% *Artemisia*-ZnO NP treatment compared with the 0.25% chemically synthesized ZnO NP treatment throughout storage was not fully explained by differences in acidity alone.

Antioxidant activity decreased significantly during refrigerated storage in all treatments, which is consistent with previous studies reporting a gradual decline in antioxidant activity in kefir and fortified kefir during storage [7,8]. Nevertheless, kefir supplemented with 0.25% chemically synthesized ZnO NPs also exhibited higher DPPH radical-scavenging activity than plain kefir throughout storage. This observation suggests that the higher antioxidant activity of the supplemented kefir samples was not attributable solely to *Artemisia*-derived phytochemicals. Other factors, including interactions between ZnO NPs and the kefir matrix, changes in physicochemical properties during storage, or other matrix-related effects, may have also influenced the measured antioxidant activity. Further studies involving phytochemical quantification and appropriate mechanistic analyses in the final kefir product are needed to clarify the relative contribution of these factors.

### 3.3. Microbiological Analysis Results

**Figure 6:**
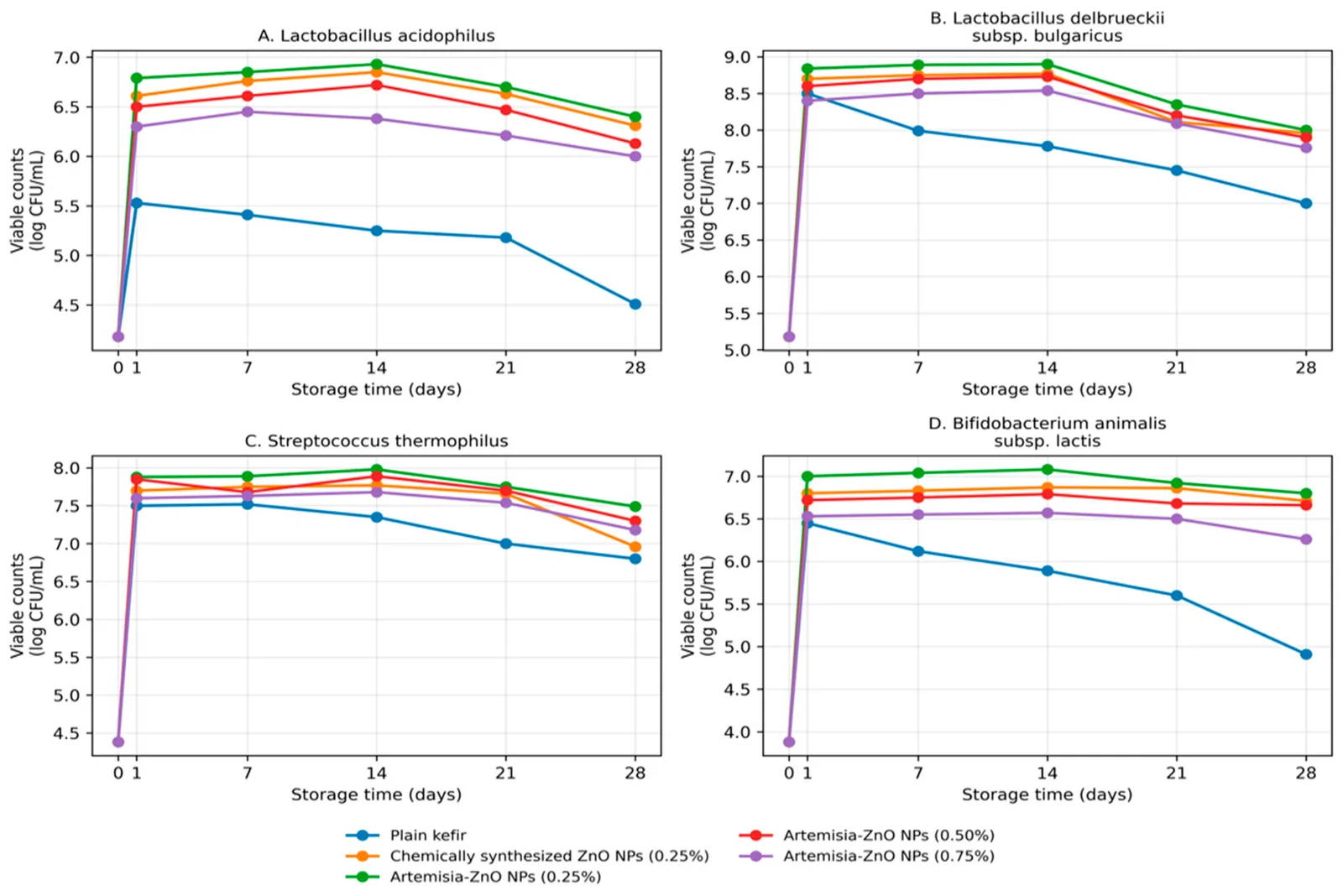
Changes in viable counts (log CFU/mL) of (**A**) *Lactobacillus acidophilus*, (**B**) *Lactobacillus delbrueckii* subsp. *bulgaricus*, (**C**) *Streptococcus thermophilus*, and (**D**) *Bifidobacterium animalis* subsp. *lactis* in plain kefir, kefir supplemented with 0.25% (*w*/*w*) chemically synthesized ZnO NPs, and kefir supplemented with *Artemisia*-ZnO NPs at 0.25%, 0.50%, and 0.75% (*w*/*w*) during refrigerated storage at 4 °C. Viable counts were determined on days 0, 1, 7, 14, 21, and 28. The treatment samples originated from a single production batch and were evaluated at the designated sampling times throughout the refrigerated storage period. Day 0 represents the post-fermentation baseline measurement before ZnO NP incorporation.

The viable counts of *Lactobacillus acidophilus*, *Lactobacillus delbrueckii* subsp. *bulgaricus*, *Streptococcus thermophilus*, and *Bifidobacterium animalis* subsp. *lactis* were monitored during 28 days of refrigerated storage (Supplementary Table S1). The bacterial counts measured at Day 0 were identical among all samples because measurements were performed after completion of the fermentation period and before the incorporation of *Artemisia*-ZnO NPs or chemically synthesized ZnO NPs. Therefore, the identical Day 0 values represent a common pre-treatment baseline. Following NP incorporation, differences in viable counts were observed among the treatment groups (*p* < 0.05), with kefir containing 0.25% *Artemisia*-ZnO NPs generally maintaining the highest viable counts, whereas plain kefir showed the greatest decline (Figure 6). During refrigerated storage, viable counts reached their highest levels during the early stage (Days 1–14), after which they gradually declined toward the end of storage. Nevertheless, all nanoparticle supplemented samples retained significantly higher probiotic viable counts than the plain kefir control, particularly those containing 0.25% *Artemisia*-ZnO NPs (Figure 6).

The higher probiotic viable counts observed in kefir supplemented with *Artemisia*-ZnO NPs may be associated with bioactive compounds present in the *Artemisia*-derived nanoparticle system. Previous studies have reported that such phytochemicals may support probiotic growth and inhibit competing microorganisms [33,37]. These compounds may also contribute to a more favorable environment for probiotic survival during refrigerated storage; however, their specific contribution to the higher viable counts observed in the present study was not directly determined. Compared with 0.25% chemically synthesized ZnO NPs, the green-synthesized *Artemisia*-ZnO NPs consistently maintained higher viable counts for all probiotic species throughout storage, indicating an association between the type of ZnO NPs and probiotic survival under the conditions tested.

Although refrigerated storage at 4 °C generally limits bacterial proliferation, a slight increase in viable counts was observed for some probiotic strains during the early storage period (Days 1–14). This increase represents a higher number of recoverable viable cells during the early storage period; however, the underlying mechanism was not directly determined in the present study. Thereafter, viable counts gradually decreased in all treatments, which is consistent with the typical behavior of fermented dairy products during refrigerated storage, as reported in previous studies [4,10,14]. Factors such as increasing acidity, nutrient depletion, and accumulation of organic acids have been proposed to contribute to the subsequent decline in bacterial survival during storage [4,10,14].

At the higher nanoparticle concentration of 0.75%, probiotic viable counts did not increase further and, in several cases, were slightly lower than those observed with the 0.25% and 0.50% treatments. This concentration-dependent variation is consistent with previous studies reporting that the effects of ZnO NPs on probiotic viability can differ according to nanoparticle concentration [14,38]. Similarly, a previous study on ZnO NP-fortified probiotic yogurt reported differences in probiotic viability among supplementation levels [20], further indicating that probiotic responses can vary with the concentration of ZnO NPs.

In the present study, the 0.25% *Artemisia*-ZnO NP treatment resulted in the highest probiotic viable counts among the three *Artemisia*-ZnO NP concentrations tested under refrigerated storage conditions. Notably, the viable counts of *Lactobacillus acidophilus* and *Bifidobacterium animalis* subsp. *lactis* in plain kefir declined below the commonly recommended minimum level of 10^6^ CFU/mL (6 log CFU/mL) for probiotic products by Day 28 of storage [39], whereas kefir supplemented with *Artemisia*-ZnO NPs maintained counts above this threshold. This observation indicates that the supplemented formulations maintained higher recoverable viable counts than plain kefir under the storage conditions tested.

Comparable strategies have previously been employed to enhance probiotic stability in fermented dairy products. Microencapsulation improved the survival of *Bifidobacterium animalis* subsp. *lactis* in kefir, while green-synthesized selenium nanorods increased the viable counts of *Bifidobacterium animalis* subsp. *lactis* and *Lactobacillus rhamnosus* in yogurt [10,40]. These findings, together with the present results, indicate that different formulation approaches can be associated with differences in probiotic viability during refrigerated storage. Overall, the present findings show that 0.25% *Artemisia*-ZnO NPs was associated with the highest viable counts among the tested *Artemisia*-ZnO NP concentrations under the conditions of the present study. However, further investigations are required to determine the mechanisms underlying the observed differences in probiotic viability and the interactions between green-synthesized ZnO NPs and probiotic bacteria. Long-term stability, zinc exposure, bioavailability, and safety should also be evaluated before any conclusions regarding their application in fermented dairy products can be established.

### 3.4. Physicochemical Analysis Results

The color parameters of kefir varied across the treatment groups and storage times (Table 2). The a* values became progressively more negative during storage, indicating a shift toward the green direction. This change was generally more pronounced with the increase in concentration of *Artemisia*-ZnO NPs, with the lowest value being observed in samples containing 0.75% *Artemisia*-ZnO NPs on Day 28 (−3.15 ± 0.09). This concentration-related response suggests that compounds associated with the green-synthesized NPs may have contributed to the color characteristics of the fortified kefir.

Clear differences were also observed in lightness (L*). While L* increased in plain kefir and samples containing 0.25% chemically synthesized ZnO NPs, it decreased slightly in samples containing *Artemisia*-ZnO NPs. On Day 28, L* reached 80.41 ± 0.04 with 0.25% chemically synthesized ZnO NPs, whereas values of 74.60 ± 0.02–74.22 ± 0.08 were observed with the higher concentrations of *Artemisia*-ZnO NPs. The lower lightness is likely to result from plant-derived compounds associated with the green-synthesized NPs and their interactions with milk constituents. Changes in the color characteristics of kefir following incorporation of plant-derived functional ingredients have also been reported previously [41].

However, the changes in L* cannot be attributed only to *Artemisia*-derived compounds. Lightness in fermented dairy products is strongly influenced by light scattering within the matrix and can therefore change with alterations in protein organization, particle distribution, and water mobility. In addition, during storage, structural changes such as micro-syneresis may modify the organization of the kefir matrix and consequently its optical properties. Moreover, interactions between ZnO NPs and food proteins can also alter the organization of matrix components [15,32]. Thus, the observed L* behavior likely reflects the combined effects of nanoparticle type, *Artemisia*-derived surface compounds, and structural changes occurring during refrigerated storage.

The b* values also changed during storage, with more negative values being generally observed at higher concentrations within the *Artemisia*-ZnO NP treatments. However, the response was not strictly concentration-dependent, as the lowest value on Day 28 was observed in kefir supplemented with 0.50% *Artemisia*-ZnO NPs (−2.96 ± 0.11) rather than 0.75% (−2.89 ± 0.08). This suggests that the color response was affected not only by the amount of incorporated NPs but also by their interactions with the heterogeneous kefir matrix. Overall, the changes in a*, L*, and b* show that the optical characteristics of kefir were influenced by a combination of nanoparticle properties and storage-related changes in the dairy matrix.

Both pH and titratable acidity changed during refrigerated storage, with clear differences among samples (Table 2). In plain kefir, the decrease in pH during refrigerated storage was accompanied by a corresponding increase in titratable acidity, reflecting continued acidification during storage. Following ZnO NP incorporation, all NP-containing treatments showed an increase in pH during the early storage period, with greater increases observed at higher concentrations of *Artemisia*-ZnO NPs. Thereafter, pH gradually decreased during subsequent storage. By Day 28, plain kefir exhibited the lowest pH (4.35 ± 0.08) and highest titratable acidity (1.02 ± 0.10 g lactic acid/100 g), whereas samples containing *Artemisia*-ZnO NPs maintained comparatively higher pH and lower acidity. The intermediate response observed with 0.25% chemically synthesized ZnO NPs further highlights differences associated with the presence and type of ZnO NPs in the progression of acidification during refrigerated storage (Table 2).

The higher pH and lower titratable acidity observed in samples containing *Artemisia*-ZnO NPs were associated with a lower degree of post-acidification during refrigerated storage. The observed changes may reflect interactions between the ZnO-containing formulations and components of the kefir matrix [32]; however, the specific mechanisms underlying these changes were not directly investigated in the present study. The concentration-dependent response observed with *Artemisia*-ZnO NPs also indicates that factors related to the nanoparticle formulation, rather than ZnO alone, may have contributed to the observed changes. Plant-derived surface compounds associated with the green-synthesized nanoparticles could also be responsible for the observed response. Previous studies on ZnO NP-fortified yogurt similarly reported changes in the physicochemical and microbiological characteristics of fermented dairy products during storage [21,22]. Overall, these findings suggest an association between *Artemisia*-ZnO NP supplementation and the observed changes in post-acidification during refrigerated storage under the conditions of the present study.

The viscosity of all kefir samples increased during the early storage period, reached maximum values on Day 14 and subsequently declined moderately toward Day 28. This increase was more pronounced with the increase in concentrations in the *Artemisia*-ZnO NP treatments. On Day 14, samples containing 0.75% *Artemisia*-ZnO NPs reached 520.09 ± 20.04 mPa·s, compared with 300.52 ± 9.08 mPa·s in plain kefir, and remained comparatively more viscous at the end of storage (Table 2). The higher viscosity is likely to reflect changes in the organization and interactions of proteins and other structural components within the kefir matrix following NP incorporation. The rheological properties of fermented dairy products are closely associated with protein–protein interactions and the organization of the gel network, and the incorporated ingredients can further modify these structural interactions [42]. The subsequent decrease after Day 14 was likely associated with storage-related rearrangement of the protein network, increasing acidity, and syneresis, which alter gel integrity and rheological behavior [43]. Supporting the influence of ZnO NPs on fermented dairy structure, a previous study on ZnO-fortified yogurt reported significant modifications in textural and physicochemical properties following ZnO incorporation [22].

Protein content increased slightly but significantly during refrigerated storage, with higher values observed in kefir supplemented with higher concentrations of *Artemisia*-ZnO NPs (Table 2). The increase may be primarily related to a concentration effect associated with slight moisture loss through syneresis during storage. This moisture loss could result in higher measured protein levels. In addition, interactions between ZnO NPs and milk proteins, including casein micelles and whey proteins, may have contributed to the observed differences through surface adsorption or electrostatic interactions [31,32]. The phytochemicals associated with *Artemisia*-ZnO NPs may also influence their interaction with the milk protein matrix, although this possibility was not directly evaluated in the present study. Similar changes in protein content and physicochemical properties have previously been reported in kefir enriched with plant-derived functional ingredients [41,44]. Furthermore, ZnO NPs have been reported to interact with food proteins and influence the structural characteristics of protein-based food systems [21,31]. Under the post-fermentation refrigerated storage conditions evaluated in the present study, the higher protein content observed in *Artemisia*-ZnO NP-supplemented kefir may therefore reflect a concentration effect associated with moisture loss during storage, together with interactions between the NPs and milk proteins.

The total solids content increased significantly during refrigerated storage, with a greater increase observed as the concentration of *Artemisia*-ZnO NPs increased. By Day 28, total solids reached 11.78 ± 0.04% in plain kefir compared with 12.12 ± 0.09% in samples containing 0.75% *Artemisia*-ZnO NPs, while samples containing chemically synthesized ZnO NPs showed an intermediate value (Table 2).

The gradual increase in total solids during storage could be attributed to whey separation (syneresis) and the associated concentration of solid constituents within the remaining kefir matrix. Structural rearrangement of the protein gel network during refrigerated storage may influence water distribution and water-holding capacity and therefore contribute to the observed changes in total solids [43]. The slightly higher values in samples containing *Artemisia*-ZnO NPs could also reflect the additional dry matter introduced by NP incorporation and associated plant-derived compounds.

The effect of added functional ingredients on the composition and physicochemical characteristics of kefir has also been documented in formulations containing plant-derived ingredients [41,44]. Overall, the observed differences in total solids are consistent with changes occurring during post-fermentation refrigerated storage and may reflect both storage-related moisture loss and the additional dry matter introduced by NP incorporation. However, because the NPs were incorporated after fermentation, the present findings do not support conclusions regarding their effect on the fermentation process.

### 3.5. Sensory Analysis Results

**Figure 7:**
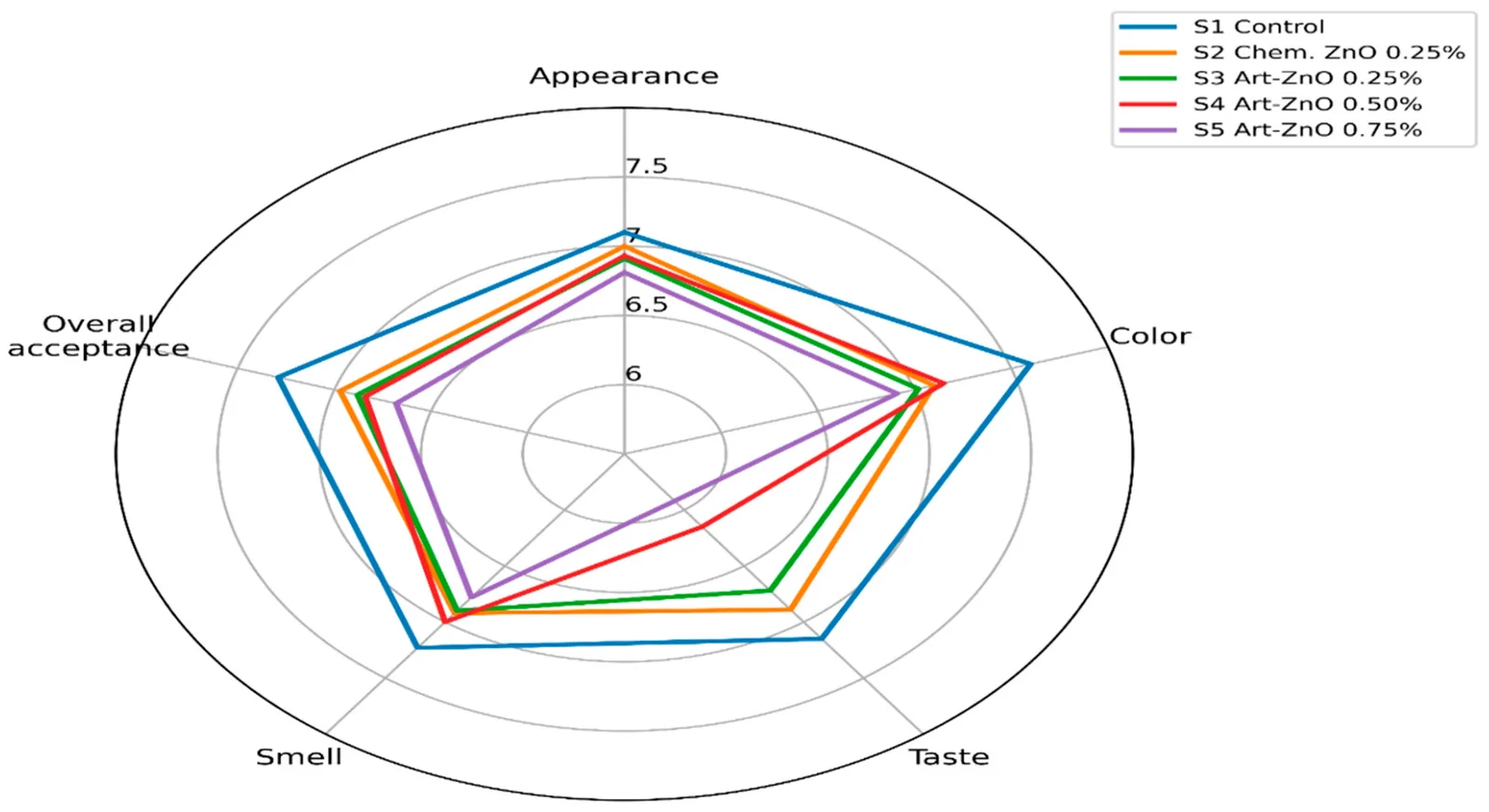
Radar plot showing the sensory profiles of plain kefir, kefir supplemented with 0.25% (*w*/*w*) chemically synthesized ZnO NPs, and kefir supplemented with *Artemisia*-ZnO NPs at concentrations of 0.25%, 0.50%, and 0.75% (*w*/*w*). Sensory attributes (appearance, color, flavor, odor, and overall acceptability) were evaluated using a 9-point hedonic scale, ranging from 1 (dislike extremely) to 9 (like extremely). Values represent the mean sensory scores of the panelists at the end of the refrigerated storage period (Day 28).

Sensory evaluation results revealed differences among the kefir samples for all evaluated attributes (Supplementary Table S2; Figure 7). Plain kefir received the highest scores for appearance (7.10 ± 0.14), color (7.60 ± 0.11), flavor (7.15 ± 0.20), odor (7.23 ± 0.13), and overall acceptability (7.29 ± 0.15). Kefir supplemented with 0.25% chemically synthesized ZnO NPs showed slightly lower sensory scores than plain kefir but remained highly acceptable across all evaluated attributes. Similarly, kefir containing 0.25% *Artemisia*-ZnO NPs demonstrated only a modest reduction in sensory scores relative to the control and showed sensory characteristics broadly comparable to those of the 0.25% chemically synthesized ZnO NP treatment.

Within the *Artemisia*-ZnO NP treatments, increasing the concentration from 0.25% to 0.50% and 0.75% was associated with lower flavor and overall acceptability scores, while appearance, color, and odor showed only slight variations among treatments. The 0.75% *Artemisia*-ZnO NP treatment exhibited the lowest ratings for appearance (6.81 ± 0.27), color (6.91 ± 0.15), flavor (5.92 ± 0.19), odor (6.78 ± 0.11), and overall acceptability (6.68 ± 0.15). Flavor was the most affected attribute, suggesting that higher concentrations of *Artemisia*-ZnO NPs may have contributed to sensory changes that reduced overall acceptability.

Taken together, the present findings showed that sensory scores differed significantly among the tested *Artemisia*-ZnO NP concentrations (*p* < 0.05), while kefir supplemented with 0.25% *Artemisia*-ZnO NPs showed sensory characteristics similar to those observed with the 0.25% chemically synthesized ZnO NP treatment. The influence of the concentration of functional ingredients on kefir sensory properties has also been reported in the literature. Kefir supplemented with a plant-derived functional ingredient at lower concentrations maintained sensory scores comparable to the control, whereas the sensory response varied among supplementation levels [41]. Similarly, sensory evaluation of yogurt fortified with different concentrations of ZnO NPs demonstrated that the level of ZnO NP incorporation influenced product acceptability, with an appropriate supplementation level providing more favorable overall characteristics [21]. These findings support the importance of optimizing the concentration of *Artemisia*-ZnO NPs to balance their technological or functional benefits with desirable sensory properties.

Although the present study demonstrated beneficial effects of *Artemisia*-ZnO NPs on probiotic viable counts and selected physicochemical properties of kefir, the actual zinc content and zinc release behavior in the final product were not determined. Therefore, the dietary zinc exposure resulting from consumption of ZnO NP-supplemented kefir could not be quantified. Based on the formulation, the 0.25% (*w*/*w*) ZnO treatment corresponds to approximately 2.5 g ZnO per kg of kefir. As elemental zinc accounts for approximately 80.3% of ZnO by mass, this represents a nominal addition of approximately 2.0 g elemental Zn per kg of kefir, equivalent to approximately 400 mg Zn per 200 g serving. This nominal amount is substantially higher than the dietary zinc Average Requirements (ARs) reported by EFSA, which range from 6.2 to 10.2 mg/day for women and from 7.5 to 12.7 mg/day for men, depending on dietary phytate intake [45], and also exceeds the tolerable upper intake level of 25 mg/day for adults established by the Scientific Committee on Food (SCF) [46]. However, this upper intake level refers to total chronic zinc intake rather than a permitted zinc concentration in a specific food, and the nominal amount calculated from the formulation does not represent the amount necessarily released from the nanoparticles, bioaccessible, or absorbed from the kefir matrix. Therefore, the ZnO concentration used in this study should be considered an experimental concentration for evaluating nanoparticle–matrix interactions and probiotic stability rather than a food-relevant intake level. Future studies should quantify total and released zinc in the final product and assess zinc bioaccessibility and bioavailability to enable a more meaningful evaluation of dietary exposure and safety.

A further limitation is that *Artemisia absinthium* L. is known to contain thujone, a bioactive compound associated with toxicological concerns upon excessive exposure [47]. Thujone content was not determined in the present study because its quantitative analysis was not included in the experimental design. Therefore, no definitive conclusion regarding thujone exposure or safety of the final kefir product can be made. Future investigations should quantify α- and β-thujone levels in *Artemisia-*derived nanoparticle formulations and the final kefir product, using an appropriate validated analytical method, and assess the resulting exposure in relation to applicable food safety requirements.

## 4. Conclusions

The incorporation of *Artemisia*-ZnO NPs at concentrations of 0.25%, 0.50%, and 0.75%, as well as 0.25% chemically synthesized ZnO NPs, was associated with differences in the microbiological, physicochemical, structural, and sensory characteristics of kefir during refrigerated storage. FESEM observations revealed aggregate-like structures within the ZnO-containing kefir matrix, while EDX detected Zn and O among the major elements; however, these findings alone do not definitively establish the identity or origin of the observed structures or the detected Zn. Particle size analysis showed a heterogeneous distribution dominated by submicron aggregates, and FTIR indicated spectral changes consistent with the ZnO-containing formulations. The 0.25% *Artemisia*-ZnO NP formulation showed the highest probiotic viable counts among the *Artemisia*-ZnO NP concentrations tested, with maximum viable counts of 6.93, 8.90, 7.98, and 7.08 log CFU/mL for *Lactobacillus acidophilus*, *Lactobacillus delbrueckii* subsp. *bulgaricus*, *Streptococcus thermophilus*, and *Bifidobacterium animalis* subsp. *lactis*, respectively. For antioxidant activity, kefir supplemented with 0.25% *Artemisia*-ZnO NPs exhibited the highest DPPH radical-scavenging activity compared with plain kefir and kefir supplemented with 0.25% chemically synthesized ZnO NPs, reaching 38.52 ± 0.16%. The higher DPPH response may reflect the combined effects of the *Artemisia*-ZnO NP system and the kefir matrix; however, the specific contribution of *Artemisia*-derived phytochemicals cannot be established because their concentrations were not quantified in the final kefir, and differences in sample pH may also have contributed to the observed DPPH response. Higher *Artemisia*-ZnO NP concentration was associated with higher viscosity, reaching 520.09 ± 20.04 mPa·s at 0.75%, together with reduced post-acidification and slight increases in protein and total solids. However, higher concentrations were associated with lower sensory acceptance.

Overall, the incorporation of *Artemisia*-ZnO NPs affected the structural and physicochemical characteristics of kefir. The 0.25% *Artemisia*-ZnO NP formulation provided the most favorable balance of probiotic viability and sensory acceptability and exhibited the highest DPPH radical-scavenging activity among the formulations evaluated for antioxidant activity. However, this overall assessment was qualitative and not based on a formal multi-criterion optimization. Nevertheless, the present study was conducted under controlled laboratory conditions and did not quantify zinc exposure or evaluate the gastrointestinal fate or bioavailability of ZnO NPs, in vivo safety, or thujone content following incorporation into kefir. Therefore, the present findings are insufficient to establish the suitability of *Artemisia*-ZnO NPs as a functional ingredient for direct application in fermented dairy products. Further studies addressing these aspects, together with larger scale sensory evaluation, quantitative assessment of phytochemicals in the final kefir formulation, and further evaluation of factors influencing DPPH measurements, are required to assess their safety and potential application in fermented dairy products.

## Figures and Tables

**Table 1:** DPPH radical-scavenging activity (%) of kefir supplemented with 0.25% (*w*/*w*) *Artemisia*-ZnO NPs and 0.25% (*w*/*w*) chemically synthesized ZnO NPs during refrigerated storage.

Sample	Storage Time					
	Day 0	Day 1	Day 7	Day 14	Day 21	Day 28
1	20.00 ± 0.15 ^Aa^	20.30 ± 0.18 ^Bc^	19.55 ± 0.23 ^Cc^	19.13 ± 0.37 ^Dc^	19.01 ± 0.26 ^Ec^	18.45 ± 0.18 ^Fc^
2	20.00 ± 0.15 ^Aa^	27.20 ± 0.21 ^Bb^	26.93 ± 0.33 ^Cb^	26.24 ± 0.18 ^Db^	26.01 ± 0.50 ^Eb^	25.10 ± 0.25 ^Fb^
3	20.00 ± 0.15 ^Aa^	38.52 ± 0.16 ^Ba^	38.13 ± 0.19 ^Ca^	38.05 ± 0.23 ^Da^	37.10 ± 0.40 ^Ea^	36.82 ± 0.37 ^Fa^

Sample identification: 1 = plain kefir; 2 = kefir supplemented with chemically synthesized ZnO NPs (0.25%); 3 = kefir supplemented with *Artemisia*-ZnO NPs (0.25%). Values are expressed as means ± SD (*n* = 3 analytical replicates). Different uppercase letters within the same row indicate significant differences among storage times within the same treatment, whereas different lowercase letters within the same column indicate significant differences among treatments at the same storage time according to Duncan’s multiple range test (*p* < 0.05). Day 0 represents the baseline measurement obtained after completion of the fermentation period (24 h) and before ZnO NP incorporation; therefore, the identical Day 0 values shown across treatment groups represent a common pre-treatment baseline.

**Table 2:** Physicochemical properties [color (a*, L* and b*), pH, titratable acidity, viscosity, protein content and total solids] of kefir supplemented with 0.25%, 0.50%, or 0.75% (*w*/*w*) *Artemisia*-ZnO NPs compared with plain kefir and kefir supplemented with 0.25% (*w*/*w*) chemically synthesized ZnO NPs during 28 days of storage.

Sample	Storage Time					
	Day 0	Day 1	Day 7	Day 14	Day 21	Day 28
	Color parameters: a* value					
1	−2.50 ± 0.12 ^Fa^	−2.55 ± 0.00 ^Ee^	−2.62 ± 0.07 ^De^	−2.70 ± 0.12 ^Ce^	−2.80 ± 0.02 ^Be^	−2.86 ± 0.05 ^Ae^
2	−2.50 ± 0.12 ^Fa^	−2.60 ± 0.08 ^Ed^	−2.70 ± 0.04 ^Dd^	−2.75 ± 0.10 ^Cd^	−2.83 ± 0.03 ^Bd^	−2.90 ± 0.12 ^Ad^
3	−2.50 ± 0.12 ^Fa^	−2.65 ± 0.08 ^Ec^	−2.79 ± 0.04 ^Dc^	−2.81 ± 0.10 ^Cc^	−2.89 ± 0.01 ^Bc^	−2.96 ± 0.10 ^Ac^
4	−2.50 ± 0.12 ^Fa^	−2.81 ± 0.03 ^Db^	−2.88 ± 0.01 ^Cb^	−2.90 ± 0.00 ^Cb^	−2.97 ± 0.07 ^Bb^	−3.01 ± 0.02 ^Ab^
5	−2.50 ± 0.12 ^Fa^	−2.95 ± 0.05 ^Ea^	−2.97 ± 0.05 ^Da^	−2.99 ± 0.23 ^Ca^	−3.10 ± 0.05 ^Ba^	−3.15 ± 0.09 ^Aa^
Color parameters: L* value						
1	75.00 ± 0.02 ^Aa^	75.63 ± 0.05 ^Aa^	77.69 ± 0.04 ^Ba^	77.63 ± 0.05 ^Ba^	77.36 ± 0.21 ^Ba^	77.16 ± 0.18 ^Ba^
2	75.00 ± 0.02 ^Aa^	77.90 ± 0.00 ^Bb^	77.91 ± 0.03 ^Ba^	80.15 ± 0.01 ^Cb^	80.26 ± 0.04 ^Cb^	80.41 ± 0.04 ^Cb^
3	75.00 ± 0.02 ^Aa^	75.16 ± 0.01 ^Aa^	75.00 ± 0.01 ^Ab^	74.89 ± 0.05 ^Bc^	74.92 ± 0.05 ^Ac^	74.60 ± 0.02 ^Cc^
4	75.00 ± 0.02 ^Aa^	74.95 ± 0.06 ^Aa^	74.76 ± 0.06 ^Bb^	74.77 ± 0.02 ^Bc^	74.68 ± 0.09 ^Cc^	74.39 ± 0.05 ^Dc^
5	75.00 ± 0.02 ^Aa^	74.60 ± 0.10 ^Bc^	74.68 ± 0.21 ^Bb^	74.35 ± 0.09 ^Cd^	74.21 ± 0.01 ^Cc^	74.22 ± 0.08 ^Cc^
Color parameters: b* value						
1	−1.56 ± 0.06 ^Da^	−1.58 ± 0.01 ^Dc^	−1.60 ± 0.00 ^Cc^	−1.64 ± 0.07 ^Bc^	−1.88 ± 0.01 ^Ac^	−1.90 ± 0.06 ^Ac^
2	−1.56 ± 0.06 ^Ca^	−0.91 ± 0.02 ^Ee^	−0.95 ± 0.02 ^De^	−1.50 ± 0.05 ^Cd^	−1.61 ± 0.05 ^Be^	−1.75 ± 0.05 ^Ae^
3	−1.56 ± 0.06 ^Ca^	−0.95 ± 0.13 ^Ed^	−0.99 ± 0.05 ^Ed^	−1.15 ± 0.08 ^De^	−1.69 ± 0.01 ^Bd^	−1.81 ± 0.20 ^Ad^
4	−1.56 ± 0.06 ^Fa^	−1.66 ± 0.02 ^Eb^	−1.77 ± 0.10 ^Db^	−2.09 ± 0.12 ^Cb^	−2.28 ±0.06 ^Bb^	−2.96 ± 0.11 ^Ab^
5	−1.56 ± 0.06 ^Ea^	−2.40 ± 0.08 ^Da^	−2.45 ± 0.02 ^Da^	−2.56 ± 0.09 ^Ca^	−2.60 ± 0.01 ^Ba^	−2.89 ± 0.08 ^Aa^
Titratable acidity						
1	0.70 ± 0.02 ^Aa^	0.76 ± 0.09 ^Ba^	0.82 ± 0.10 ^Ca^	0.89 ± 0.02 ^Da^	0.95 ± 0.06 ^Ea^	1.02 ± 0.10 ^Fa^
2	0.70 ± 0.02 ^Aa^	0.72 ± 0.05 ^Ab^	0.78 ± 0.11 ^Bb^	0.84 ± 0.05 ^Cb^	0.90 ± 0.05 ^Db^	0.96 ± 0.08 ^Eb^
3	0.70 ± 0.02 ^Aa^	0.70 ± 0.03 ^Ac^	0.74 ± 0.09 ^Bc^	0.79 ± 0.07 ^Cc^	0.84 ± 0.10 ^Dc^	0.89 ± 0.05 ^Ec^
4	0.70 ± 0.02 ^Aa^	0.68 ± 0.02 ^Bd^	0.72 ± 0.01 ^Cd^	0.76 ± 0.09 ^Dd^	0.81 ± 0.07 ^Ed^	0.86 ± 0.09 ^Fd^
5	0.70 ± 0.02 ^Aa^	0.66 ± 0.10 ^Be^	0.70 ± 0.05 ^Ce^	0.74 ± 0.05 ^De^	0.78 ± 0.04 ^Ee^	0.82 ± 0.07 ^Fe^
pH value						
1	4.70 ± 0.03 ^Da^	4.72 ± 0.05 ^Da^	4.55 ± 0.03 ^Ca^	4.48 ± 0.09 ^Ba^	4.42 ± 0.12 ^Ba^	4.35 ± 0.08 ^Aa^
2	4.70 ± 0.03 ^Da^	4.82 ± 0.09 ^Db^	4.70 ± 0.09 ^Cb^	4.62 ± 0.05 ^Bb^	4.56 ± 0.04 ^Bb^	4.50 ± 0.10 ^Ab^
3	4.70 ± 0.03 ^Da^	4.90 ± 0.06 ^Dc^	4.78 ± 0.05 ^Cc^	4.72 ± 0.07 ^Bc^	4.65 ± 0.09 ^Bc^	4.58 ± 0.09 ^Ac^
4	4.70 ± 0.03 ^Da^	4.98 ± 0.11 ^Dd^	4.88 ± 0.02 ^Cd^	4.82 ± 0.05 ^Bd^	4.75 ± 0.02 ^Bd^	4.68 ± 0.05 ^Ad^
5	4.70 ± 0.03 ^Da^	5.05 ± 0.04 ^De^	4.95 ± 0.06 ^Ce^	4.88 ± 0.02 ^Be^	4.82 ± 0.07 ^Be^	4.75 ± 0.07 ^Ae^
Viscosity						
1	120.05 ± 5.11 ^Aa^	200.32 ± 8.30 ^Ba^	260.08 ± 11.80 ^Ca^	300.52 ± 9.08 ^Da^	280.12 ± 11.86 ^Ea^	260.16 ± 9.50 ^Fa^
2	120.05 ± 5.11 ^Aa^	230.00 ± 10.12 ^Bb^	300.12 ± 12.68 ^Cb^	350.08 ± 15.08 ^Db^	330.51 ± 11.00 ^Eb^	310.00 ± 10.47 ^Fb^
3	120.05 ± 5.11 ^Aa^	250.14 ± 6.80 ^Bc^	340.30 ± 10.57 ^Cc^	400.46 ± 13.25 ^De^	380.08 ± 14.01 ^Ec^	360.42 ± 12.01 ^Fc^
4	120.05 ± 5.11 ^Aa^	280.34 ± 12.50 ^Bd^	380.48 ± 14.91 ^Cd^	470.20 ± 11.97 ^Dd^	450.39 ± 13.70 ^Ed^	430.23 ± 14.89 ^Fd^
5	120.05 ± 5.11 ^Aa^	300.28 ± 7.52 ^Be^	420.36 ± 18.80 ^Ce^	520.09 ± 20.04 ^De^	500.06 ± 22.05 ^Ee^	480.07 ± 13.78 ^Fe^
	Protein %					
1	3.10 ± 0.10 ^Aa^	3.11 ± 0.05 ^ABa^	3.12 ± 0.02 ^BCa^	3.12 ± 0.06 ^BCa^	3.14 ± 0.12 ^CDa^	3.15 ± 0.08 ^Da^
2	3.10 ± 0.10 ^Aa^	3.12 ± 0.09 ^ABa^	3.12 ± 0.05 ^ABa^	3.13 ± 0.01 ^BCa^	3.15 ± 0.10 ^CDb^	3.16 ± 0.11 ^Db^
3	3.10 ± 0.10 ^Aa^	3.14 ± 0.04 ^Bb^	3.15 ± 0.10 ^BCb^	3.15 ± 0.04 ^BCb^	3.16 ± 0.07 ^Cc^	3.18 ± 0.03 ^Dc^
4	3.10 ± 0.10 ^Aa^	3.17 ± 0.01 ^Bc^	3.18 ± 0.05 ^Cc^	3.18 ± 0.11 ^Cc^	3.20 ±0.05 ^Dd^	3.24 ± 0.05 ^Ed^
5	3.10 ± 0.10 ^Aa^	3.20 ± 0.12 ^Bd^	3.20 ± 0.08 ^Bd^	3.24 ± 0.15 ^Ce^	3.25 ± 0.09 ^Ce^	3.28 ± 0.05 ^De^
Total solid%						
1	11.50 ± 0.09 ^Aa^	11.60 ± 0.05 ^Ba^	11.66 ± 0.10 ^Ca^	11.70 ± 0.01 ^Da^	11.77 ± 0.01 ^Ea^	11.78 ± 0.04 ^Fa^
2	11.50 ± 0.09 ^Aa^	11.70 ± 0.02 ^Bb^	11.78 ± 0.05 ^Cb^	11.79 ± 0.05 ^Cb^	11.82 ± 0.05 ^Db^	11.90 ± 0.01 ^Eb^
3	11.50 ± 0.09 ^Aa^	11.72 ± 0.09 ^Bb^	11.80 ± 0.08 ^Cbc^	11.88 ± 0.01 ^Dc^	11.90 ± 0.06 ^Ec^	11.94 ± 0.05 ^Fc^
4	11.50 ± 0.09 ^Aa^	11.75 ± 0.12 ^Bb^	11.83 ± 0.09 ^Cc^	11.92 ± 0.09 ^Dd^	11.98 ± 0.13 ^Ed^	12.09 ± 0.00 ^Fd^
5	11.50 ± 0.09 ^Aa^	11.90 ± 0.09 ^Bc^	11.91 ± 0.04 ^Cd^	11.99 ± 0.04 ^De^	12.09 ± 0.07 ^Ee^	12.12 ± 0.09 ^Fe^

L* represents lightness (0 = black, 100 = white); a* represents the color axis from green (−) to red (+); b* represents the color axis from blue (−) to yellow (+). Viscosity is expressed as mPa·s (equivalent to cP); titratable acidity as g lactic acid/100 g; protein and total solids as % (*w*/*w*). Values are expressed as means ± SD (*n* = 3 analytical replicates). Different uppercase letters within the same row indicate significant differences among storage times for each treatment (*p* < 0.05). Different lowercase letters within the same column indicate significant differences among treatments at the same storage time according to Duncan’s multiple range test (*p* < 0.05). Sample identification: 1 = plain kefir; 2 = kefir supplemented with chemically synthesized ZnO NPs (0.25%); 3 = kefir supplemented with *Artemisia*-ZnO NPs (0.25%); 4 = kefir supplemented with *Artemisia*-ZnO NPs (0.50%); 5 = kefir supplemented with *Artemisia*-ZnO NPs (0.75%). Day 0 represents the baseline measurement obtained after completion of the fermentation period (24 h) and before ZnO NP incorporation; therefore, the identical Day 0 values shown across treatment groups represent a common pre-treatment baseline.

## Data Availability

The original contributions presented in this study are included in the article/Supplementary Material. Further inquiries can be directed to the corresponding author.

## Supplementary Materials

The following supporting information can be downloaded at: https://www.mdpi.com/article/10.3390/foods15183271/s1, Table S1. Effect of 0.25% (*w*/*w*) chemically synthesized ZnO NPs and 0.25%, 0.50%, or 0.75% (*w*/*w*) Artemisia-ZnO NPs on probiotic viable counts (log CFU/mL) in kefir samples during 28 days of refrigerated storage; Table S2. Sensory attributes of kefir supplemented with 0.25%, 0.50%, or 0.75% (*w*/*w*) Artemisia-ZnO NPs compared with plain kefir and kefir supplemented with 0.25% (*w*/*w*) chemically synthesized ZnO NPs; Figure S1. Laser diffraction particle size distribution of kefir supplemented with 0.25% Artemisia-ZnO NPs; Figure S2. Laser diffraction particle size distribution of kefir supplemented with 0.25% chemically synthesized ZnO NPs.
