# Supplementary material for: Comparative Evaluation of Artemisia-ZnO NPs and Chemically Synthesized ZnO NPs in Kefir: Effects on Quality and Probiotic Viability
Source: Foods. 2026 Sep 16;15(18):3271. doi: 10.3390/foods15183271 (PMC13606887; doi:10.3390/foods15183271)
Supplement: Supplementary file 1 [file foods-15-03271-s001.zip › foods-4538599-supplementary.pdf]

Supplementary Material:

Supplementary Table S1. Effect of 0.25% (w/w) chemically synthesized ZnO NPs and 0.25%, 0.50%, or 0.75% (w/w) *Artemisia*-ZnO NPs on probiotic viable counts (log CFU/mL) in kefir samples during 28 days of refrigerated storage.

		Storage time					
Probiotic	Sample	Day 0	Day 1	Day 7	Day 14	Day 21	Day 28
Lactobacillus acidophilus	1	4.18 ± 0.02^{Aa}	5.53 ± 0.00^{Ca}	5.41 ± 0.06^{Ea}	5.25 ± 0.01^{Fa}	5.18 ± 0.07^{Da}	4.51 ± 0.02^{Ba}
	2	4.18 ± 0.02^{Aa}	6.61 ± 0.05^{Cd}	6.76 ± 0.03^{Ed}	6.85 ± 0.06^{Fd}	6.63 ± 0.01^{Dd}	6.31 ± 0.00^{Bd}
	3	4.18 ± 0.02^{Aa}	6.79 ± 0.09^{De}	6.85 ± 0.04^{Ee}	6.93 ± 0.02^{Fe}	6.70 ± 0.03^{Ce}	6.40 ± 0.01^{Be}
	4	4.18 ± 0.02^{Aa}	6.50 ± 0.04^{Dc}	6.61 ± 0.05^{Ec}	6.72 ± 0.09^{Fc}	6.47 ± 0.04^{Cc}	6.13 ± 0.05^{Bc}
	5	4.18 ± 0.02^{Aa}	6.30 ± 0.09^{Db}	6.45 ± 0.02^{Fb}	6.38 ± 0.03^{Eb}	6.21 ± 0.21^{Cb}	6.00 ± 0.06^{Bb}
Lactobacillus delbrueckii subsp. *bulgaricus*	1	5.18 ± 0.05^{Aa}	8.50 ± 0.06^{Fb}	7.99 ± 0.01^{Ea}	7.78 ± 0.05^{Da}	7.45 ± 0.00^{Ca}	7.00 ± 0.01^{Ba}
	2	5.18 ± 0.05^{Aa}	8.70 ± 0.02^{Dd}	8.75 ± 0.02^{Ed}	8.77 ± 0.00^{Ed}	8.11 ± 0.05^{Cb}	7.96 ± 0.05^{Bd}
	3	5.18 ± 0.05^{Aa}	8.84 ± 0.02^{De}	8.89 ± 0.00^{DEe}	8.90 ± 0.01^{De}	8.35 ± 0.00^{Cd}	8.00 ± 0.00^{Be}
	4	5.18 ± 0.05^{Aa}	8.60 ± 0.00^{Dc}	8.70 ± 0.01^{Ec}	8.73 ± 0.03^{Fc}	8.20 ± 0.04^{Cc}	7.90 ± 0.00^{Bc}
	5	5.18 ± 0.05^{Aa}	8.40 ± 0.05^{Da}	8.50 ± 0.00^{Eb}	8.54 ± 0.00^{Fb}	8.09 ± 0.02^{Cb}	7.76 ± 0.04^{Bb}
Streptococcus thermophilus	1	4.38 ± 0.02^{Aa}	7.50 ± 0.06^{Ea}	7.52 ± 0.01^{Fa}	7.35 ± 0.05^{Da}	7.00 ± 0.00^{Ca}	6.80 ± 0.01^{Ba}
	2	4.38 ± 0.02^{Aa}	7.70 ± 0.02^{Dc}	7.75 ± 0.02^{Ed}	7.77 ± 0.00^{Fc}	7.66 ± 0.05^{Cc}	6.96 ± 0.05^{Bb}
	3	4.38 ± 0.02^{Aa}	7.88 ± 0.05^{De}	7.89 ± 0.00^{Ee}	7.98 ± 0.01^{Fe}	7.75 ± 0.00^{Ce}	7.49 ± 0.02^{Be}
	4	4.38 ± 0.02^{Aa}	7.85 ± 0.00^{Ed}	7.68 ± 0.01^{Dc}	7.89 ± 0.03^{Fd}	7.70 ± 0.08^{Cd}	7.30 ± 0.00^{Bd}
	5	4.38 ± 0.02^{Aa}	7.60 ± 0.04^{Db}	7.63 ± 0.00^{Eb}	7.68 ± 0.00^{Fb}	7.54 ± 0.02^{Cb}	7.18 ± 0.01^{Bc}
Bifidobacterium animalis subsp. *lactis*	1	3.88 ± 0.02^{Aa}	6.45 ± 0.06^{Fa}	6.12 ± 0.01^{Ea}	5.89 ± 0.05^{Da}	5.60 ± 0.00^{Ca}	4.91 ± 0.01^{Ba}
	2	3.88 ± 0.02^{Aa}	6.80 ± 0.02^{Cd}	6.83 ± 0.02^{Dd}	6.87 ± 0.00^{Ed}	6.86 ± 0.05^{Ed}	6.71 ± 0.05^{Bd}
	3	3.88 ± 0.02^{Aa}	7.00 ± 0.01^{De}	7.04 ± 0.05^{Ee}	7.08 ± 0.01^{Fe}	6.92 ± 0.00^{Ce}	6.80 ± 0.02^{Be}
	4	3.88 ± 0.02^{Aa}	6.72 ± 0.00^{Dc}	6.75 ± 0.01^{Ec}	6.79 ± 0.03^{Fc}	6.68 ± 0.08^{Cc}	6.66 ± 0.00^{Bc}
	5	3.88 ± 0.02^{Aa}	6.53 ± 0.04^{Db}	6.55 ± 0.00^{Eb}	6.57 ± 0.00^{Fb}	6.50 ± 0.02^{Cb}	6.26 ± 0.01^{Bb}

Values are expressed as mean ± SD (n = 3 analytical replicates). Different uppercase letters within the same row indicate significant differences among storage times for each treatment ($p < 0.05$). Different lowercase letters within the same column indicate significant differences among treatments at the same storage time according to Duncan's multiple range test ($p < 0.05$). Sample identification**:** 1 = Plain kefir; 2 = Kefir supplemented with chemically synthesized ZnO NPs (0.25%); 3 = Kefir supplemented with *Artemisia*-ZnO NPs (0.25%); 4 = Kefir supplemented with *Artemisia*-ZnO NPs (0.50%); 5 = Kefir supplemented with *Artemisia*-ZnO NPs (0.75%). Day 0 represented the baseline measurement obtained after completion of the fermentation period (24 h) and before ZnO NPs incorporation; therefore, the identical Day 0 values shown across treatment groups represent a common pre-treatment baseline

Supplementary Table S2. Sensory attributes of kefir supplemented with 0.25%, 0.50%, or 0.75% (w/w) *Artemisia*-ZnO NPs compared with plain kefir and kefir supplemented with 0.25% (w/w) chemically synthesized ZnO NPs.

Samples	Appearance	Color	Flavor	Odor	Overall acceptance
1	7.10±0.14^{e}	7.60±0.11^{e}	7.15±0.20^{e}	7.23±0.13^{e}	7.29±0.15^{e}
2	7.00±0.16^{d}	7.10±0.14^{c}	6.89±0.15^{d}	6.92±0.14^{c}	6.97±0.21^{d}
3	6.91±0.18^{b}	7.02±0.15^{b}	6.72±0.16^{c}	6.90±0.15^{b}	6.88±0.50^{c}
4	6.93±0.12^{c}	7.15±0.10^{d}	6.15±0.28^{b}	7.00±0.05^{d}	6.84±0.16^{b}
5	6.81±0.27^{a}	6.91±0.15^{a}	5.92±0.19^{a}	6.78±0.11^{a}	6.68±0.15^{a}

Samples: 1 = plain kefir; 2 = kefir supplemented with 0.25% chemically synthesized ZnO NPs; 3 = kefir supplemented with 0.25% *Artemisia*-ZnO NPs; 4 = kefir supplemented with 0.50% *Artemisia*-ZnO NPs; 5 = kefir supplemented with 0.75% *Artemisia*-ZnO NPs. Values are expressed as mean ± SD (n = 3 analytical replicates). Different lowercase letters within the same column indicate significant differences among samples according to Duncan's multiple range test ($p < 0.05$). Sensory attributes were evaluated using a 9-point hedonic scale, where 1 = dislike extremely, 2 = dislike very much, 3 = dislike moderately, 4 = dislike slightly, 5 = neither like nor dislike, 6 = like slightly, 7 = like moderately, 8 = like very much, and 9 = like extremely.

Supplementary Figure S1. Laser diffraction particle size distribution of kefir supplemented with 0.25% *Artemisia*-ZnO NPs.

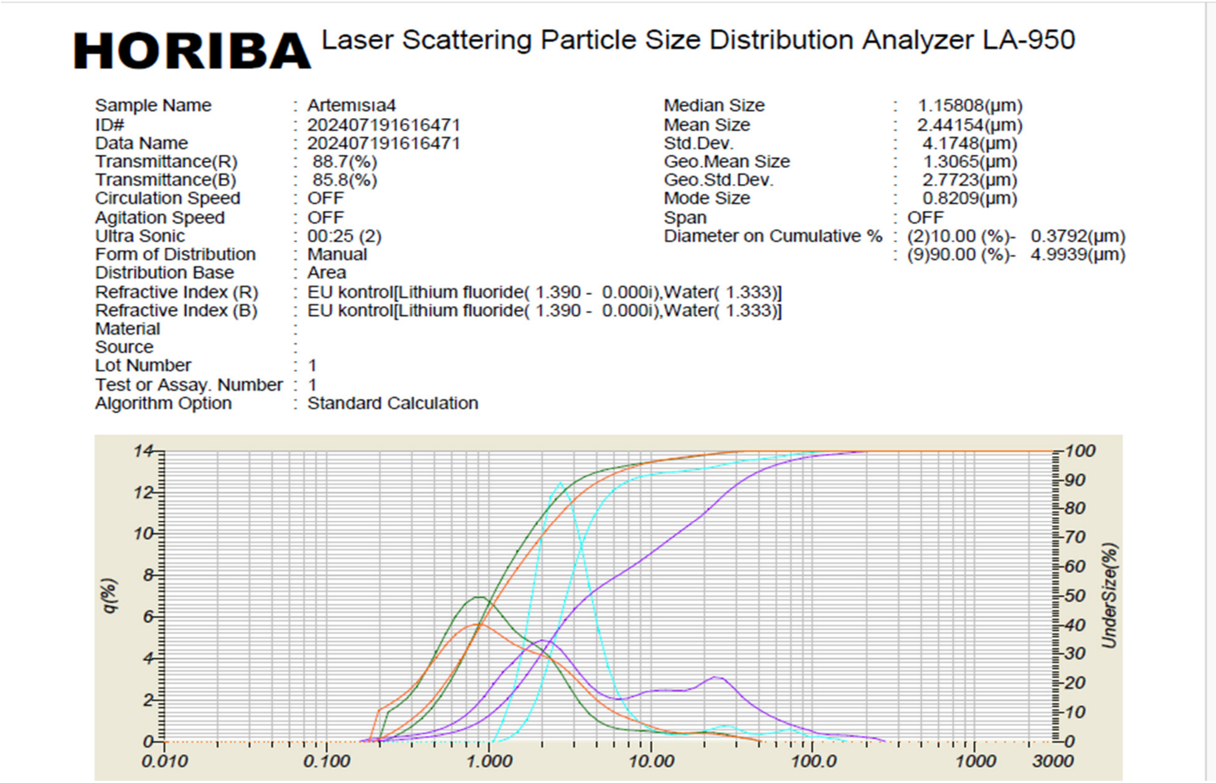

Supplementary Figure S2. Laser diffraction particle size distribution of kefir supplemented with 0.25%. chemically synthesized ZnO NPs.

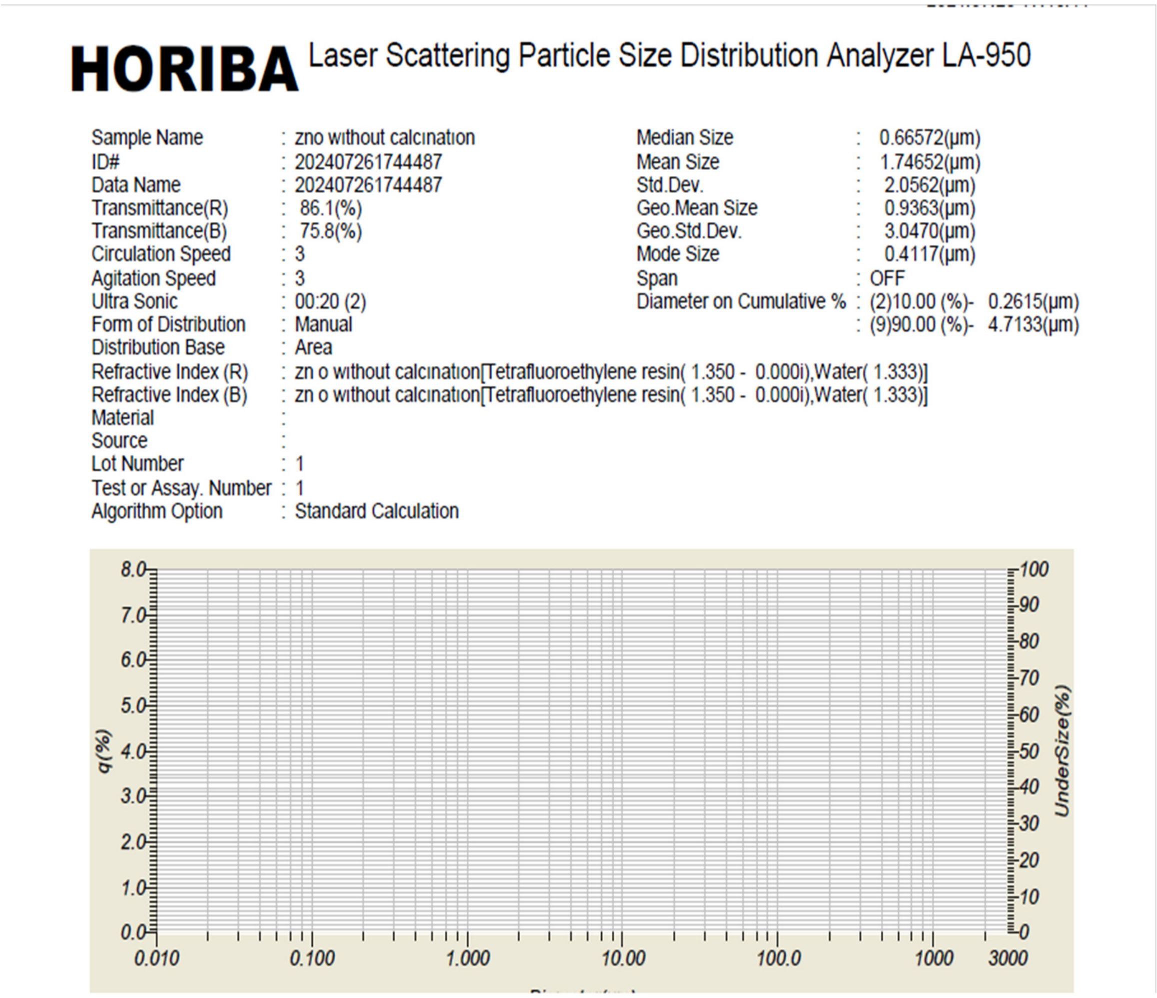

HORIBA Laser Scattering Particle Size Distribution Analyzer LA-950

Sample Name	: zno without calcination	Median Size	: 0.66572(µm)
ID#	: 202407261744487	Mean Size	: 1.74652(µm)
Data Name	: 202407261744487	Std.Dev.	: 2.0562(µm)
Transmittance(R)	: 86.1(%)	Geo.Mean Size	: 0.9363(µm)
Transmittance(B)	: 75.8(%)	Geo.Std.Dev.	: 3.0470(µm)
Circulation Speed	: 3	Mode Size	: 0.4117(µm)
Agitation Speed	: 3	Span	: OFF
Ultra Sonic	: 00:20 (2)	Diameter on Cumulative %	: (2)10.00 (%)- 0.2615(µm)
Form of Distribution	: Manual		: (9)90.00 (%)- 4.7133(µm)
Distribution Base	: Area		
Refractive Index (R)	: zn o without calcination[Tetrafluoroethylene resin(1.350 - 0.000i),Water(1.333)]		
Refractive Index (B)	: zn o without calcination[Tetrafluoroethylene resin(1.350 - 0.000i),Water(1.333)]		
Material	:		
Source	:		
Lot Number	: 1		
Test or Assay. Number	: 1		
Algorithm Option	: Standard Calculation		
